# TMUB1 Inhibits BRL-3A Hepatocyte Proliferation by Interfering with the Binding of CAML to Cyclophilin B through its TM1 Hydrophobic Domain

**DOI:** 10.1038/s41598-018-28339-4

**Published:** 2018-07-02

**Authors:** Xiang Lan, Hangwei Fu, Guangyao Li, Wei Zeng, Xia Lin, Yuanxin Zhu, Menggang Liu, Ping Chen

**Affiliations:** Department of Hepatobiliary Surgery, The Third Affiliated Hospital, The Third Military Medical University (Army medical university), Chongqing, China

## Abstract

Transmembrane and ubiquitin-like domain-containing 1 (Tmub1) encodes a protein (TMUB1) containing an ubiquitin-like domain and plays a negative regulatory role during hepatocyte proliferation, but its mechanism in this process is still unknown. Here, TMUB1 interfered with the binding of calcium-modulating cyclophilin ligand (CAML) to cyclophilin B, which may represent a key role in the negative regulatory process of TMUB1 in hepatocyte proliferation. Co-immunoprecipitation assays in rat BRL-3A cells confirmed the interaction between TMUB1 and CAML; significant regulation of the influx of Ca2+ ([Ca2+]i) and hepatocyte proliferation occurred following TMUB1 overexpression or knockout. Deletion of the TM1 hydrophobic domain of TMUB1 completely abolished this interaction and led to loss of TMUB1’s regulatory effects on cytological behavior. Furthermore, overexpression of TMUB1 completely abolished the interaction between CAML and its downstream protein cyclophilin B, which can act upstream of calcineurin by increasing [Ca2+]i during cell proliferation. Taken together, our results indicate that TMUB1 regulates BRL-3A hepatocyte proliferation by interacting with CAML and further interferes with the binding of CAML to cyclophilin B to decrease cellular [Ca2+]i.

## Introduction

Tmub1 encodes a shuttle protein, TMUB1/HOPS, that contains two hydrophobic transmembrane domains and one ubiquitin-like domain (UBL)^1,2^. UBL proteins or molecules are divided into the following separate classes: ubiquitin-like protein modifiers (ULMs), which share significant sequence similarity with ubiquitin through the E1/E2/E3 protein systems, and ubiquitin domain-containing proteins (UDPs), which directly recognize polyubiquitinated proteins through the 26S proteasome system or escort a subset of polyubiquitinated proteins to the 26S proteasome for degradation^3–6^. Moreover, some UDPs execute functions such as membrane receptor trafficking and posttranslational modification^7–9^. TMUB1 is one of a few UDPs with many functions, mechanisms, and interactions with other proteins that remain unknown. TMUB1 plays a role in binding other proteins, facilitating the recycling of GluR2 to the plasma membrane, acting as an essential constituent of centrosome assembly during cellcycles in culture and interacting with CAML in the cytoplasm of neurons^10–13^.

CAML is a ubiquitous protein that localizes mainly to the endoplasmic reticulum (ER) and small cytoplasmic vesicles^14,15^. CAML is required for EGF receptor recycling and regulation of membrane trafficking of GABA receptor and interacts with other proteins to facilitate insertion of these proteins into the ER membrane or to stabilize their molecular structures^16–18^. Furthermore, CAML binding to cyclophilin B and calreticulinacts upstream of calcineurin by causing an influx of calcium ([Ca2+]i) and facilitates the calcium-dependent activation of nuclearfactors of activated T cells^14,19^. CAML overexpression depletes [Ca2+]i pools. Through the [Ca2+]i signaling pathway, CAML can regulate processes in breast cancer cell proliferation, B-cell activation and T-cell development^20–22^.

In our previous studies, overexpression of TMUB1 had a negative impact on hepatocyte proliferation, and TMUB1 and CAML in BRL-3A hepatocytes were co-immunoprecipitated (CO-IP)^23^, but the mechanism remained unknown. Here, we report a new function of TMUB1 that interferes with the binding of CAML to cyclophilin B to decrease [Ca2+]i.

## Results

### Tmub1 gene and protein were abundantly expressed after rat partial hepatectomy and in BRL-3A hepatocytes

Tmub1 mRNA and protein were overexpressed in residual hepatocytes after rat partial hepatectomy (PH) in our previous study^23^. Furthermore, there are three different TMUB1 isoforms, including a long isoform (lTMUB1/HOPS; 27 kDa), intermediate molecular-weight isoform (iTMUB1/HOPS; 24 kDa) and short isoform (sTMUB1/HOPS; 21 kDa). The long isoform interacts with other proteins, plays a significant role, while the intermediate and short isoform are released from the long isofrom, and shuttles between intracellular compartments^24^. So in our study, in search of the binding domain that interacted with CAML, the long isoform (lTMUB1/HOPS) was mainly identified.

**Figure 1:**
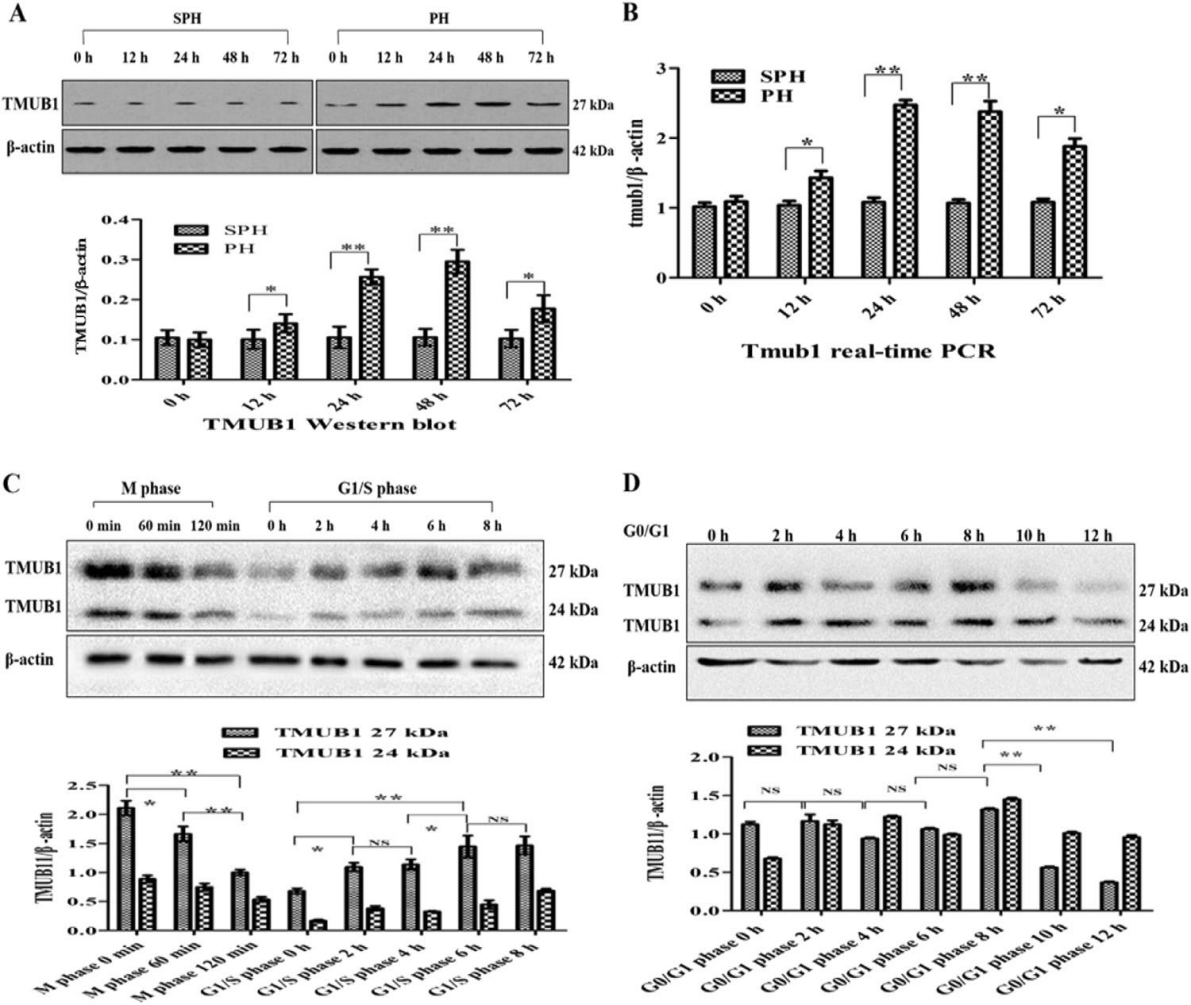
TMUB1 expression. (**A**) Western blot analysis of TMUB1 in the sham-operated (SPH) group and partial hepatectomy (PH) groupat 0, 12, 24, 48, and 72 hours. TMUB1 expression significantly increased after PH until 48 hours, and then the expression decreased gradually (n = 3, *P < 0.05, **P < 0.01, t-test) (**B**) mRNA expression of Tmub1 between the SPH and PH groups were in agreement with the protein changes in proliferative cells. (n = 3, *P < 0.05, **P < 0.01, t-test). (**C**) Western blot analysis of TMUB1 in the M phase and G1/S phase. The expression of TMUB1 significantly decreased in the M phase and increased in the G1/S phase (n = 3, *P < 0.05, **P < 0.01, ANOVA); (**D**) Western blot analysis of TMUB1 in the G0/G1 phase. The expression of TMUB1 did not change in the beginning of the G0/G1 phase (0–8 hours) but significantly decreased at the end of this phase (10 and 12 hours) (n = 3, *P < 0.05, **P < 0.01, ANOVA).

Here, we detected different expression levels of Tmub1 mRNA and protein at 0, 12, 24, 48 and 72 hours following PH. TMUB1 expression was analyzed by Western blotting in rat normal liver and regenerating liver. At 12 hours after PH, TMUB1 expression began to increase, peaked at 48 hours and then decreased at 72 hours (Fig. 1A). Consistently, Tmub1 mRNA also peaked at 48 hours after PH (Fig. 1B).

To test TMUB1 expression in BRL-3A rat hepatocytes *in vitro*, we synchronized the cell cycle at the G0/G1 boundary in serum-freemedium, in the G1/S phase with the double thymidine method and at the M phase with nocodazole. According to the length of different cell cycles, the cells were harvested at 0, 2, 4, 6, 8, 10, and 12 hours in G0/G1 after release; at 0, 2, 4, 6, and 8 hours in the G1/S phase after release; and at 0, 60, and 120 min in the M phase after release. According to flow cytometry analysis, 67.79–84.5% of BRL-3A cells were synchronized in the G0/G1 phase, 53.65–66.94% in the G1/S phase and 78.94–80.57% in the M phase (Supplementary Fig. 4A–O). The expression levels of TMUB1 gradually decreased in the M phase and increased in the G1/S phase after release (Fig. 1C). The expression levels of TMUB1 decreased at the end of the G0/G1 phase (10 and 12 hours) after release (Fig. 1D).

### TMUB1 co-precipitated and co-localized with CAML in BRL-3A hepatocellular cytoplasm

**Figure 2:**
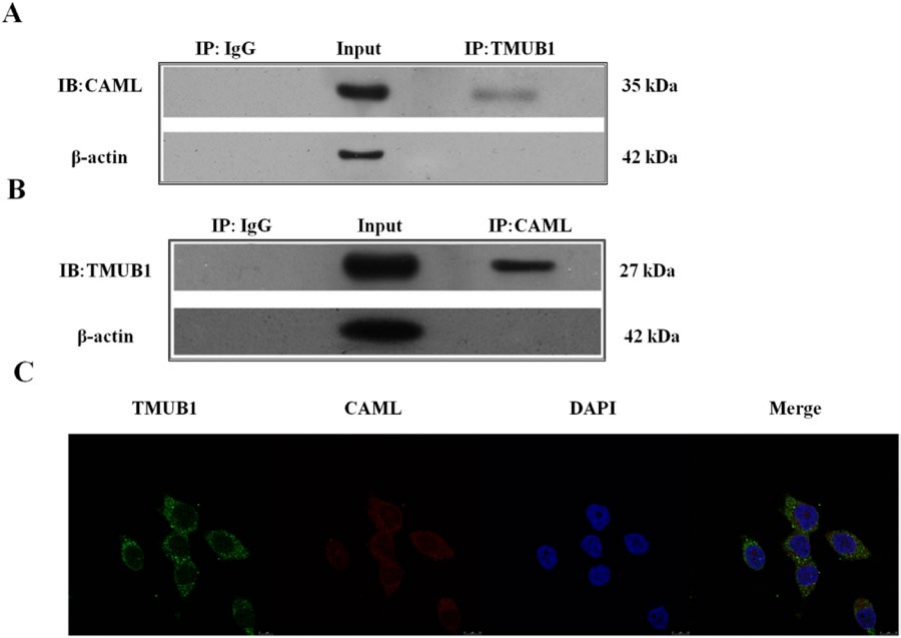
Immunoprecipitation for magnetic beads and laser scanning confocal imaging (**A**). Magnetic beads were suspended with an anti-TMUB1 antibody, and the nitrocellulose membrane was incubated with an anti-CAML antibody. (**B**) Magnetic beads were suspended with an anti-CAML antibody, and the nitrocellulose membrane was incubated with an anti-TMUB1 antibody. These two results demonstrated the co-immunoprecipitation of TMUB1 and CAML in BRL-3A hepatocyte lysis buffer. (**C**) Laser scanning confocal imaging of TMUB1 and CAML; these two proteins were co-localized to BRL-3A cell cytoplasm.

In previous studies, TMUB1 was co-precipitated and functioned with CAML in neurons and BRL-3A hepatocytes^13,23^. Here, we furtherly confirmed their relationship with two co-immunoprecipitations (IP: TMUB1, IB: CAML and IP: CAML, IB: TMUB1, Fig. 2A,B) and laser scanning confocal imaging (Fig. 2C).

### TMUB1 interacted with CAML through its C-terminal TM1 hydrophobic domain

**Figure 3:**
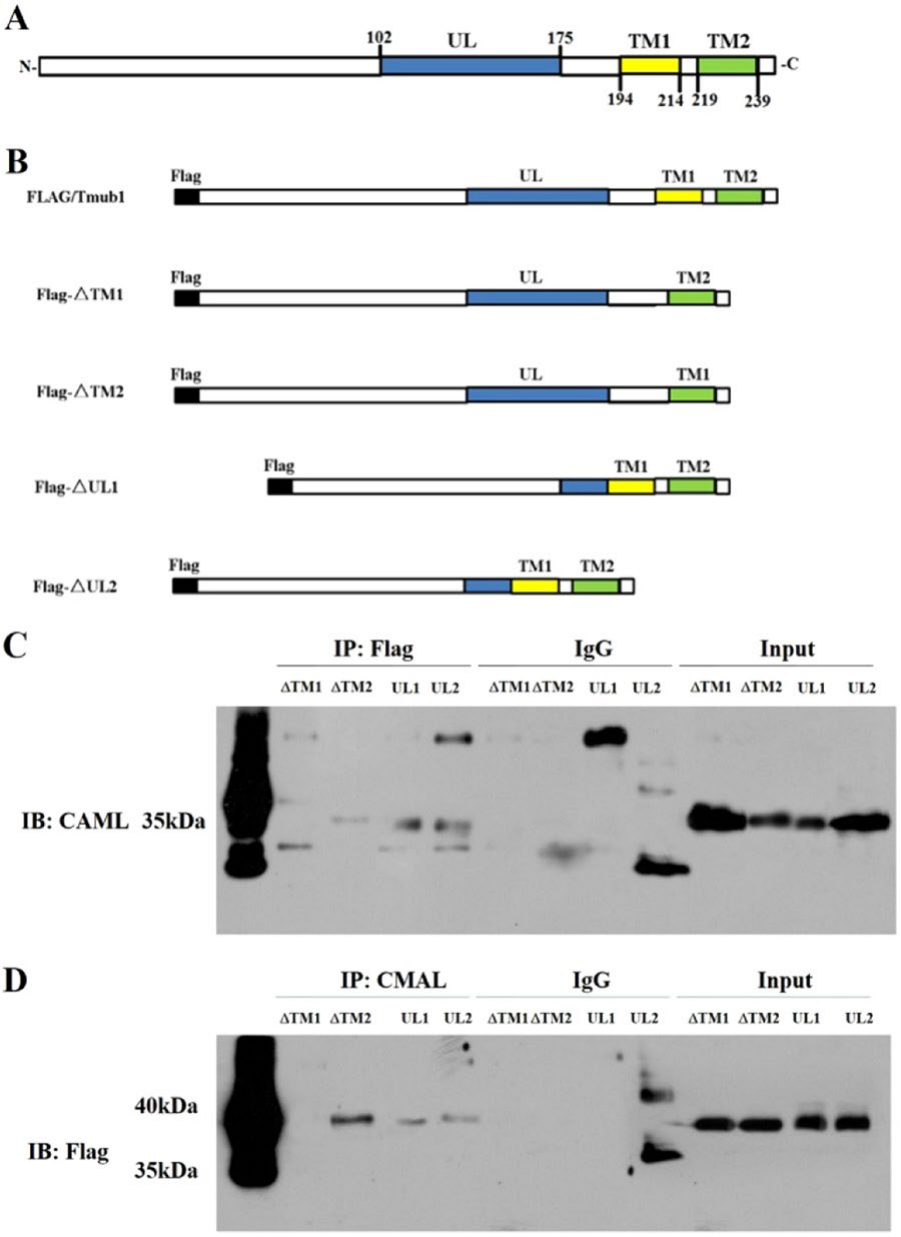
TMUB1 binding site interacts with CAML. (**A**) Domain structure of rat TMUB1. (**B**) Flag-tagged full-length TMUB1 and its mutants. Flag-ΔTM1: Flag-tagged TMUB1 lacking the TM1 domain; Flag-ΔTM2: Flag-tagged TMUB1 lacking the TM2 domain; Flag-ΔUL1: Flag-ΔTM2: Flag-tagged TMUB1 lacking the first segment of the ubiquitin-like domain; flag-ΔUL2: Flag-tagged TMUB1 lacking the second segment of the ubiquitin-like domain. (**C**) Magnetic beads were suspended with an anti-Flag antibody, and the nitrocellulose membrane was incubated with an anti-CAML antibody. Deletion of the TM1 hydrophobic domain completely abolished the interaction between TMUB1 and CAML. (**D**) Magnetic beads weresuspended withan anti-CAML antibody, and the nitrocellulose membrane was incubated with an anti-Flag antibody. This result once again confirmsthat the TM1 hydrophobic domain is the binding site of TMUB1 that interacts with CAML.

According to available bioinformatics prediction (http://www.ncbi.nlm.nih.gov/protein/AAH00936.1), the cDNA for rat TMUB1 predicts a 245-aminoacid protein containing one ubiquitin-like domain (102–175) and two C-terminal hydrophobic domains, TM1 (194–214) and TM2 (219–239) (Fig. 3A). To determine which region of TMUB1 mediates its binding to CAML, we transfected cells with an expression plasmid encoding domain mutants (Fig. 3B). Considering the long sequence of the ubiquitin-like domain and that loss of the whole domain can lead to TMUB1 instability, we divided the domain into two segments to construct the mutants (UL1 102–138 and UL2 139–175, Fig. 3B and Supplementary Gene sequence).

Co-immunoprecipitation was used to compare the binding of the mutant forms of TMUB1. Deletion of the TM1 hydrophobic domain completely abolished the interaction between TMUB1 and CAML. By contrast, mutant TMUB1 lacking TM2, UL1 and UL2 continued to associate with CAML (Fig. 3C,D). These findings are very different from our previous speculation that TMUB1 interacts with CAML through its ubiquitin-like domain, leading to the ubiquitination and degradation of CAML.

Together, these results indicated that the C-terminal TM1 hydrophobic domain of TMUB1 significantly contributes to its association with CAML.

### [Ca2+]i influx was inhibited when TMUB1 interacted with CAML

**Figure 4:**
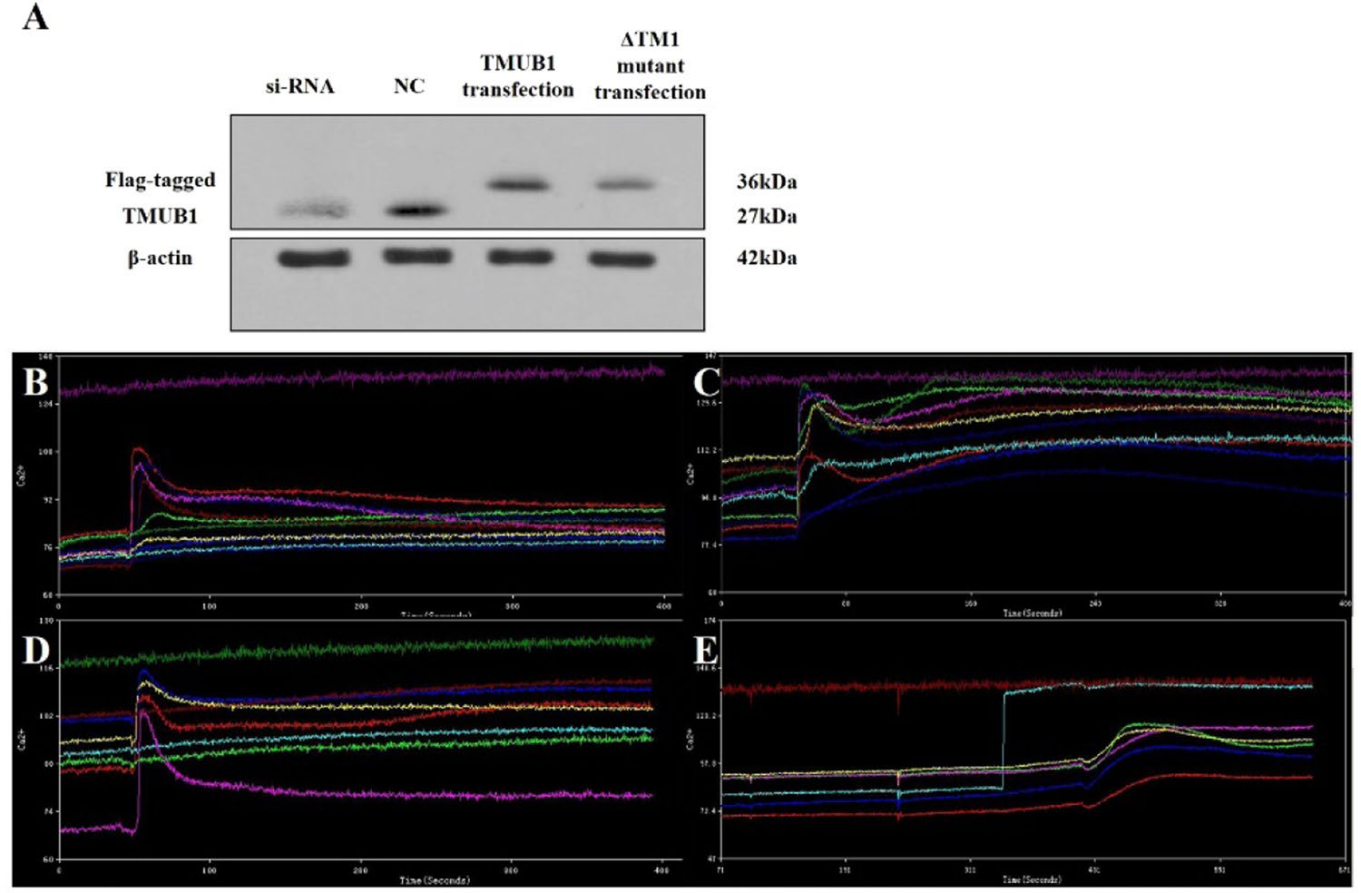
The impact of TMUB1 on [Ca2+]i in BRL-3A hepatocytes. (**A**) The effects of transfection of full-length TMUB1, its mutants and siRNA were tested by Westernblotting. BRL-3A hepatocytes were prepped for experiments on day 2 when the cells reached 50–60% confluence; the cells were then transfected with pcDNA-TMUB1-Flag, pcDNA-ΔTM1-Flag, pcDNA-control and Tmub1-siRNA. (**B**–**E**) Measurement of [Ca2+]i levels. The cells were harvested and detached with 0.25% trypsin into a cell suspension 72 hours after transfection. Images were obtained from five randomly selected fields, and at least seven cells were observed per visual field (indicated by the different colored lines). The average peak values of [Ca2+]i in BRL-3A hepatocytes transfected with pcDNA-control (**B**), Tmub1-siRNA (**C**), pcDNA-ΔTM1-Flag (**D**) and pcDNA-TMUB1-Flag (**E**) were98.3 ± 1.5 nm, 128.1 ± 3.0 nm, 103.7 ± 3.5 nm, respectively, and nearly no peak wave was observed at baseline (the average baseline was 75.6 ± 3.5). TMUB1 knockout significantly increased [Ca2+]i and lasted for a long time, while overexpression of TMUB1 significantly inhibited [Ca2+]i. Furthermore, when the interaction of TMUB1 andCAML was disturbed by deletion of the binding site (ΔTM1), [Ca2+]i in the ΔTM1 group exhibited no significant changes compared with that in the control group (F = 268.3, *P* < 0.01, ANOVA between the four groups and *P* = 0.18, LSD between the ΔTM1 group and control group).

Intracellular free calcium is a ubiquitous second messenger regulating a multitude of normal and pathogenic cellular responses, including the proliferation of hepatocytes and other cancer cells. Therefore, the upstream signaling pathways regulating the intracellular free calcium concentration may have a significant impact on hepatocyte proliferation. CAML activates [Ca2+]i influx signaling and appears to participate in Ca2+-dependent signaling initiated by the transmembrane activator and CAML interactor cell surface receptor^25^. Thus, we designed experiments to determine whether TMUB1 has an impact on the [Ca2+]i influx signaling through its interaction with CAML. The cells were transfected with an expression plasmid encoding Flag-tagged full-length TMUB1, siRNA and normal control. An expression plasmid encoding TMUB1 lacking the TM1 domain (ΔTM1) was also transfected after wild-type TMUB1 was silenced to interfere with its interaction with CAML (Fig. 4A).

The peak of [Ca2+]i was transiently detected at a slightly lower level and quickly returned to normal in the normal control group and ΔTM1 group (Fig. 4B,D). The peak of [Ca2+]i was increased and lasted for a long time after TMUB1 was silenced, while [Ca2+]i was not detected in cells overexpressing TMUB1 (Fig. 4C,E).

These results indicated that TMUB1 can decrease [Ca2+]i, and when its interaction with CAML is disturbed, the effect of TMUB1 on [Ca2+]i vanishes. Therefore, we can confirm that TMUB1 regulates [Ca2+]i through an interaction with CAML.

### Calcium concentration in primary cells after rat PH and SPH

To further illustrate the inhibitive effect of TMUB1 on the [Ca2+]i *in vivo*, we investigated the calcium concentration in primary cells at different times after rat PH and SPH. These primary cells were harvested from remnant liver tissues at 0, 12, 24, 48 and 72 hours following operation. Calcium concentration was tested by flow cytometry.

**Figure 5:**
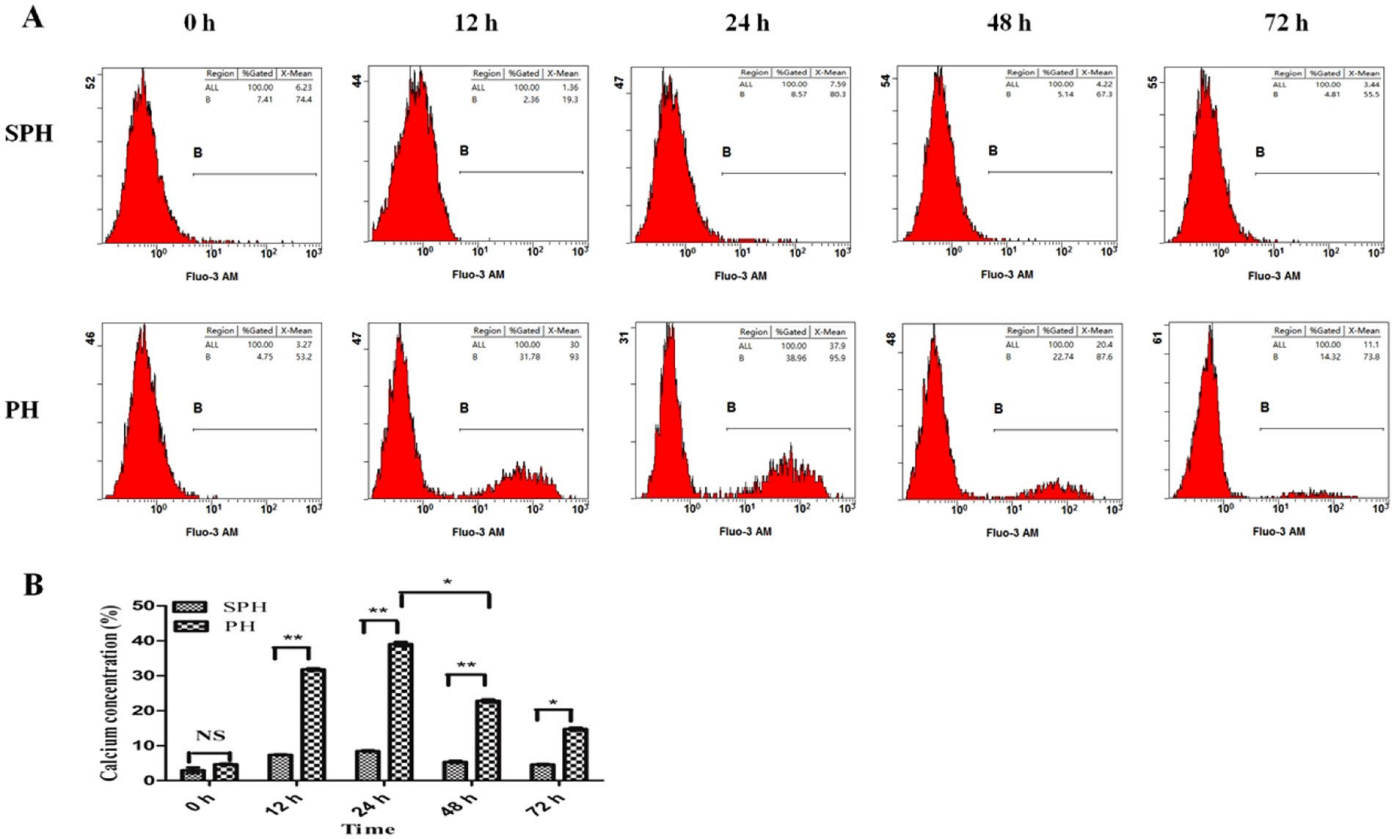
(**A**,**B**) The testing of calcium concentration in primary cells. In SPH group, the average of calcium concentration at 0, 12, 24, 48 and 72 hours was 2.99 ± 0.79, 7.39 ± 0.34, 8.46 ± 0.24, 5.54 ± 0.34 and 4.60 ± 0.22, respectively (n = 3 in each time points); In PH group, the average of calcium concentration at 0, 12, 24, 48 and 72 hours was 4.68 ± 0.23, 31.83 ± 0.33, 39.07 ± 0.62, 22.80 ± 0.43 and 14.75 ± 0.38, respectively (n = 3 in each time points). Comparing with SPH group, the calcium concentration in primary cells of PH group was significantly higher at 12, 24, 48 and 72 hours after rat hepatectomy (*P < 0.05, **P < 0.01, t-test). In PH group, the calcium concentration rapidly decreased at 48 hours after hepatectomy when the expression of TMUB1 was significantly higer, which was comfirmed by previous steps (Fig. 1A).

Calcium concentration in hepatocytes significantly increased and peaked at 12–24 hours after PH. However, following the increase of TMUB1 at 48 hours (Fig. 1A), calcium concentration rapidly decreased (Fig. 5A,B). These results signify that TMUB1 has a negative effect on intracellular calcium concentration *in vivo*. Together with these two steps, we speculate that following the regeneration of remnant rat liver tissues, the expression of TMUB1 begins to increase, inhibits the [Ca2+]i of hepatocytes through interacting with CAML and accordingly leads to the decrease of intracellular calcium concentration. Finally, TMUB1 plays a role in the preventing hyperplasia of the liver.

### TMUB1 interfered with the binding of CAML to cyclophilin B

**Figure 6:**
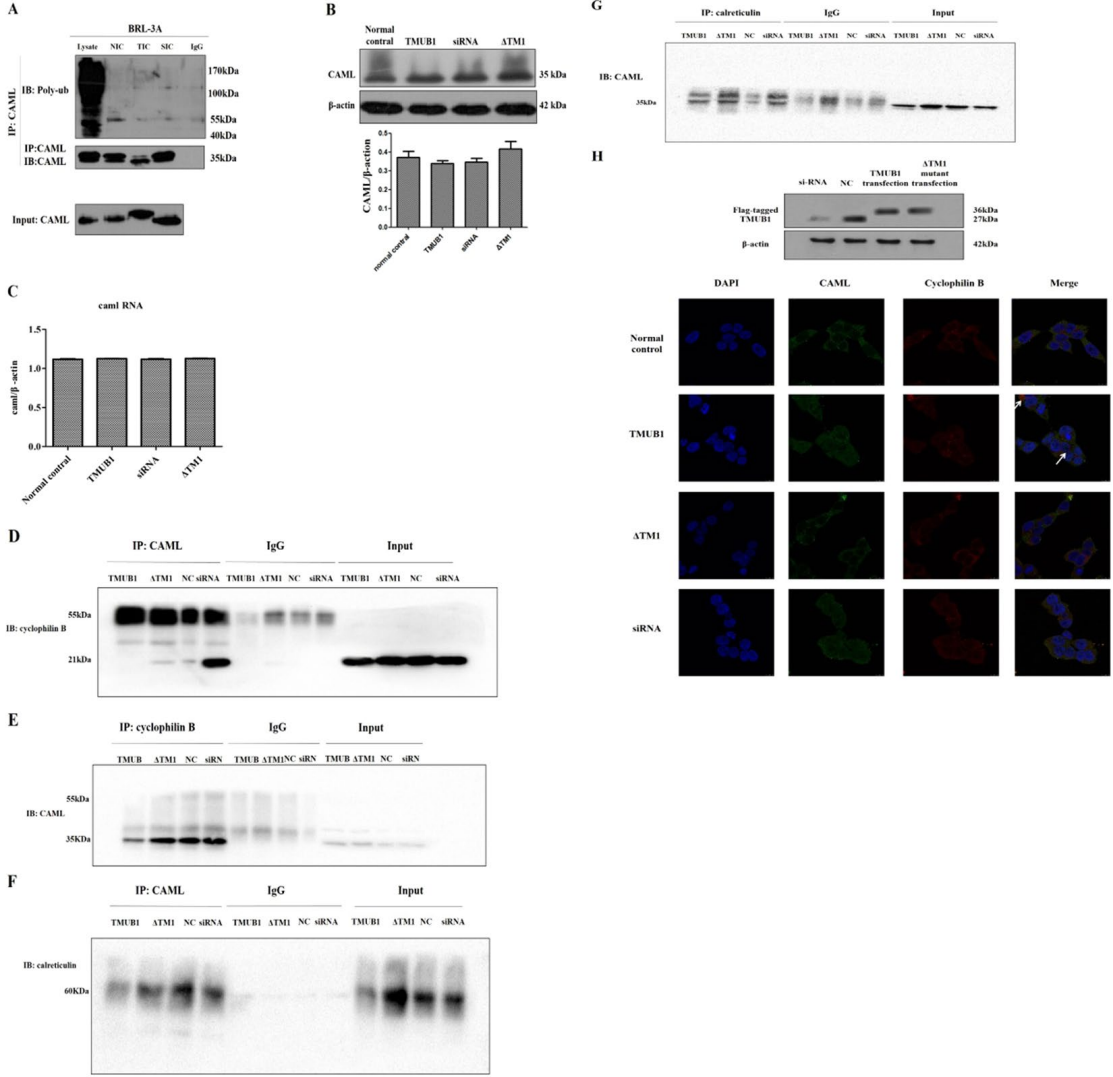
The mechanism by which TMUB1 acts on CAML (**A**). Ubiquitylation of CAML was tested after transfection of different TMUB1 mutants. Lysate: the lysate of BRL-3A hepatocytes was used as a positive control; NIC: normal control immunoprecipitation complex of CAML; TIC: full-length TMUB1-transfected immunoprecipitated complex of CAML; SIC: siRNA-transfected immunoprecipitated complex of CAML; IgG: negative control. No ubiquitylation of CAML was observed when TMUB1 was overexpressed or knocked out. (**B**) The expression of CAML did not exhibit significant changes before and after TMUB1 transfection (F = 1.467, *P* = 0.295, ANOVA). (**C**) The expression of CAML RNA did not show significant changes before and after TMUB1 transfection (F = 4.026, P > 0.05, ANOVA). (**D**) Magnetic beads were suspended with an anti-CAML antibody, and the nitrocellulose membrane was incubated with an anti-cyclophilin B antibody. Overexpression of TMUB1 abolished the interaction between CAMLand cyclophilin B; when TMUB1 interfered with CAML (ΔTM1 group), there was no effect on the interaction between CAML and cyclophilin B. TMUB1 knockout led to a close interaction between CAML and cyclophilin B. (**E**) Magnetic beads were suspended with an anti-cyclophilin B antibody, and the nitrocellulose membrane was incubated with an anti-CAML antibody. The interaction between cyclophilin B antibodies and CAML was significantly decreased after TMUB1 overexpression. (**F**) Magnetic beads were suspended with an anti-CAML antibody, and the nitrocellulose membrane was incubated with an anti-calreticulin antibody. The interaction between CAMLand calreticulin was not affected by TMUB1 overexpression or knockout. (**G**) Magnetic beads were suspended with an anti-calreticulin antibody, and the nitrocellulose membrane was incubated with an anti-CAML antibody. (**H**) Laser scanning confocal microscopy: in normal BRL-3A hepatocytes, the distribution pattern of CAML (green) was partly co-localized with cyclophilin B (red), which centered around the karyotheca, and CAML was also distributed in the cytoplasm. But CAML was separated from cyclophilin B after Tmub1 transfection (white arrows).

To investigate the mechanism through which TMUB1 acts on CAML, we designed experiments to determine this mechanism. We first speculated that TMUB1 could promote the degradation of CAML by ubiquitin through its ubiquitin-like domain. However, in this study, no ubiquitylation was observed when TMUB1 was overexpressed (Fig. 6A). The expression levels of CAML mRNA and protein did not significantly change before and after Tmub1 transfection (Fig. 6B,C).

CAML acts upstream of calcineurin by causing [Ca2+]i influx and facilitating calcium-dependent activation by binding to cyclophilin B and calreticulin^14,19^. Since TMUB1 could not regulate CAML on both transcriptional and posttranslational modification levels, we next speculated that TMUB1 regulated [Ca2+]i by disturbingthe CAML interaction with cyclophilin B or calreticulin when it bound to CAML. BRL-3A hepatocytes transfected with pcDNA-TMUB1-Flag, pcDNA-ΔTM1-Flag, pcDNA-controland Tmub1-siRNAwereharvested on day 2. The interaction of CAML with cyclophilin B or calreticulin was tested by co-immunoprecipitation. Overexpression of TMUB1 abolished the interaction between CAML and cyclophilin B, while TMUB1 knockout led to aclose interaction between CAML and cyclophilin B (Fig. 6D,E). Neither overexpression nor knockout of TMUB1 could affect the interaction between CAML and calreticulin (Fig. 6F,G). These results indicated that TMUB1 could affect the interaction between CAML and cyclophilin B. When the interaction between TMUB1 and CAML is disturbed by deletion of the TM1 domain, the effect of TMUB1 on the interaction between CAML and cyclophilin B vanishes.

To confirm the results, we observed the relationship between CAML and cyclophilin B before and after TMUB1 transfection under a laser scanning confocal microscope. CAML was separated from the ER after overexpression of TMUB1. In the three groups (pcDNA-ΔTM1-Flag, pcDNA-control and Tmub1-siRNA), the distribution pattern of CAML was consistent with that of cyclophilin B (Fig. 6H).

### TMUB1 inhibited BRL-3A hepatocyte proliferation. When the interaction between TMUB1 and CAML was disturbed, this effect on hepatocyte proliferation vanished

To investigate whether TMUB1 regulated hepatocyte proliferation through CAML, we transfected BRL-3A with different mutants and full-length TMUB1. The cells were inoculated in 96-well plates with 1000 cells/well after transfection with pcDNA-TMUB1-Flag, pcDNA-ΔTM1-Flag, and pcDNA-control. A flash, full wavelength scanning, multifunction reading instrument was used to count cells at 0, 24, 48 and 72 hours after WST-8 was added to the wells (CCK-8 methods). BRL-3A hepatocytes transfected with plasmid were prepped for EDU experiments on day 2 after inoculation in 96-well plates with 1000 cells/well.

**Figure 7:**
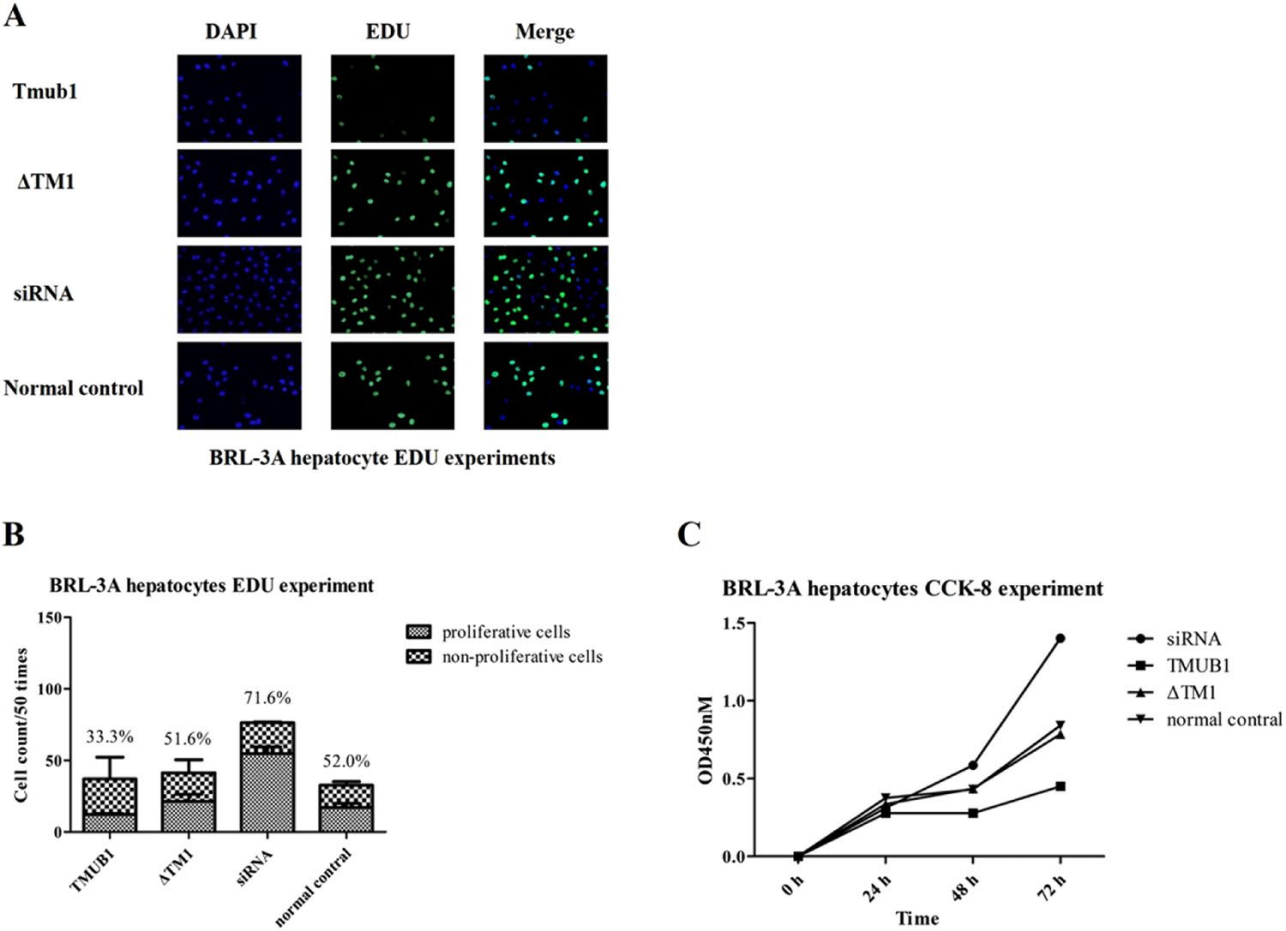
TMUB1 inhibited BRL-3A hepatocyte proliferation through aninteraction with CAML (**A**,**B**). EDU experiments in BRL-3A hepatocytes. Hepatocytes transfected with TMUB1 had the fewest cells and lowest number of proliferative cells in every well. By contrast, TMUB1 knockout resulted in significantly more proliferative cells. When the interaction between TMUB1 and CAML was disturbed, the cell proliferation rates in the ΔTM1 group were nearly the same as those in the normal control group (*X*^2^ = 53.386, *P* < 0.01, chi-square test between four groups and *X*^2^ = 1.906, *P* = 0.107, between the ΔTM1 group and control group. The transfection test is shown in Fig. 6H). **C**. CCK-8 experiment in BRL-3A hepatocytes. Absorbance was not significantly different at 0 and 24 hours (F = 1.661, *P* = 0.252, ANOVA between four groups) but was significantly different at 48 (F = 9.690, *P* < 0.01, ANOVA between four groups) and 72 hours (F = 8.934, *P* < 0.01, ANOVA between four groups). When the interaction between TMUB1 and CAML was disturbed, the absorbance in the ΔTM1 group was almost the same as that in the normal control group at 24 hours (*P* = 0.438, LSD between the ΔTM1 group and control group), 48 hours (*P* = 0.941, LSD between the ΔTM1 group and control group) and 72 hours (*P* = 0.769, LSD between the ΔTM1 group and control group).

Overexpression of TMUB1 inhibited BRL-3A hepatocyte proliferation, while TMUB1 knockout significantly promoted cell proliferation. When the interaction between TMUB1 and CAML was disturbed, the effect of TMUB1 on cell proliferation vanished (Fig. 7A–C).

## Discussion

UDPs are expected to have diverse roles in various biological processes. However, the functions of many members in this family remain unclear. TMUB1, one type of UDP, can transport other crucial proteins according to different cell cycles that play a role in DNA repair, centrosome assembly, etc.^26^. Moreover, TMUB1 acts as a functional bridge in the interaction between NPM and p19Arf^12^. Various biological processes connected to TMUB1 need to be determined.

TMUB1 is overexpressed in hepatoma cells and plays a negative regulatory role in the process of hepatocyte proliferation^23,27^. However, the mechanism and signaling pathway through which TMUB1 exertsits effects on cell biology remain unknown. Here, we introduced a novel role for TMUB1 as a negative regulatory molecule in the process of [Ca2+]i associating with CAML, an integral membrane protein that was originally identified in lymphocytes. When CAML interacts with cyclophilin B, it can act upstream of calcineurin by causing increased [Ca2+]i^19^. Overexpression of CAML could deplete [Ca2+]i stores, cause activation of [Ca2+]I and lead to activation of nuclear factor of activated T cell transcription factor (NF-AT)-dependent transcriptional activity^28^. [Ca2+]i is a ubiquitous second messenger that regulates a multitude of normal and pathogenic cellular responses, including the development of cell growth and metastasis^29,30^. Here, overexpression of TMUB1 led to decrease of [Ca2+]i and intracellular calcium concentration, interference with the interaction between CAML and cyclophilin B and inhibition of BRL-3A hepatocyte proliferation. After the binding site of TMUB1 with CAML was deleted, the effect of TMUB1 on [Ca2+]i and BRL-3A hepatocyte proliferation vanished.

UDP can directly recognize polyubiquitinated proteins by the 26S proteasome system or escort asubset of polyubiquitinated proteins to the 26S proteasome for degradation. TMUB1 is one protein in the UDP families. Because TMUB1contains a UBL domain, we speculated that TMUB1 could transfer CAML to the 26S proteasome, leading to CAML degradation. However, in this study, the expression of CAMLwas unchanged before and after TMUB1 transfection. Accordingly, ubiquitination of CAML was also unaltered after Tmub1 transfection.

Moreover, the specificity of the interaction between CAML and cyclophilin B was first verified by two-hybrid reverse-swap experiments^31^. CAML cannot interact with cyclophilin A or C. In addition, the complex of cyclophilin B with the immunosuppressant drug cyclosporin A can bindand inhibit the calcium-dependent phosphatase calcineurin^32^. Because weak binding of cyclophilins to calcineurin can be detected even in the absence of cyclosporin A, it has been proposed that cyclophilins normally participate in the regulation of calcineurin^33^. As a cyclophilin-binding protein, CAML can bind cyclophilin B present at the “calciosome” (a subspecialized region of the ER involved incalcium homeostasis)^19^. This interaction enhances the binding of cyclophilins to calcineurin and leads to activation of NF-AT and further regulates intracellular calcium releaseor generates the signal responsible for opening plasma membrane calcium channels. This finding is in accordance with our results; when the interaction between CAML and cyclophilin B was abolished by overexpression of TMUB1, [Ca2+]i was decreased.

**Figure 8:**
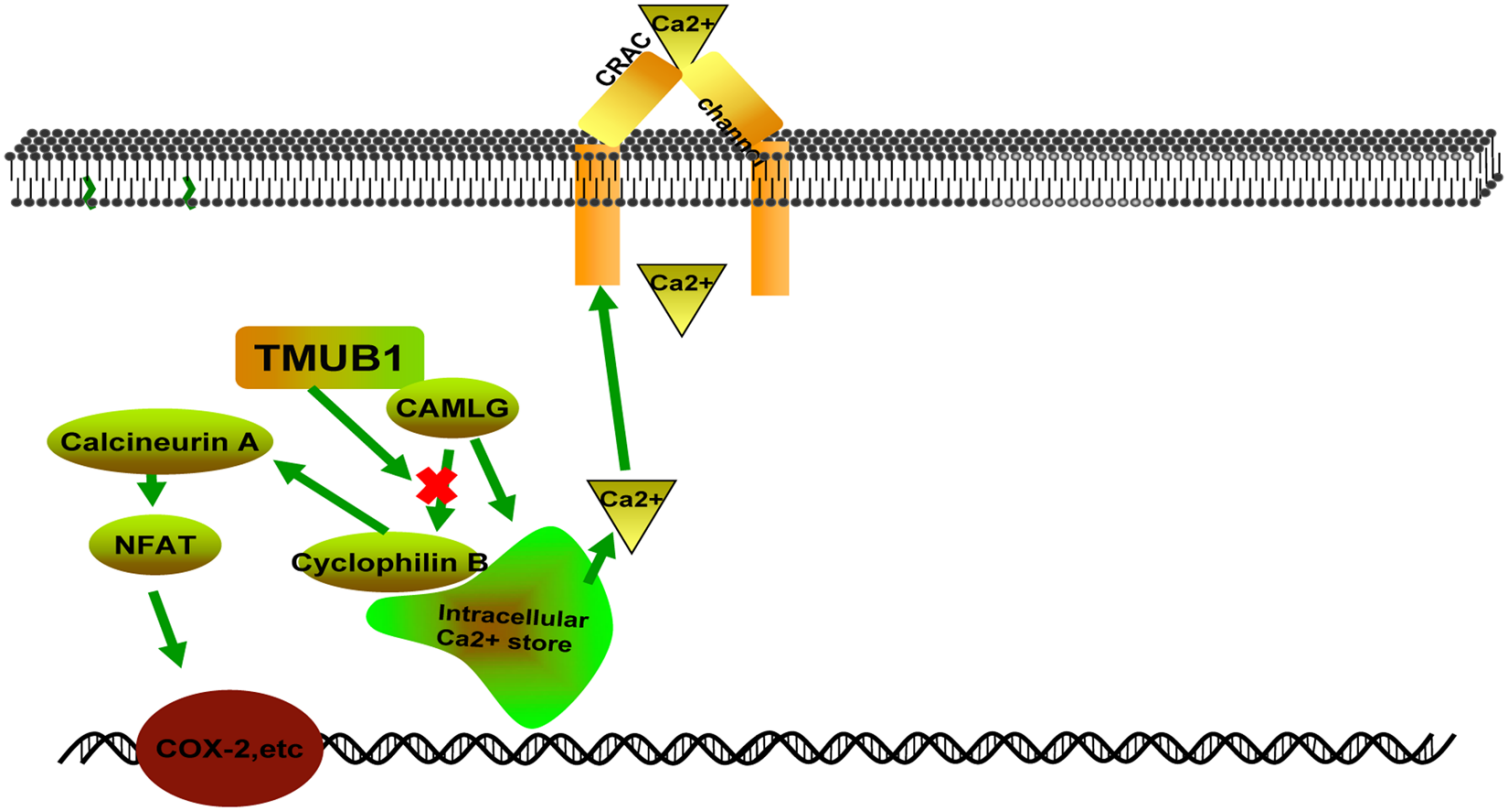
TMUB1 acts upstream of the Ca2+ signaling pathway.

Moreover, CAML directly participates in Ca2+ -dependent signaling initiatedby the transmembrane activator and CAML interactor cell surface receptor through colocalizing with sarcoplasmic/ER calcium/ATPase-2 and calreticulin at membrane-bound cytosolic vesicles^14^. However, in our results, overexpression of TMUB1 seems to have no effect on the interaction between CAML and calreticulin. TMUB1 acts upstream of calcineurin through its interaction with CAML (TMUB1/CAML/cyclophilin B/calcineurin/NF-AT pathways) and may be one of the ways that TMUB1 inhibits cell proliferation (Fig. 8).

We previously confirmed that the expression of TMUB1 in liver tissues after rat PH is consistent with the regularity of liver regeneration. DNA begins to duplicate at 12–16 hours after PH, and the residual liver grows to 45–50% of the original liver volume at 24 hours after PH^34^. At 12 hours after PH, TMUB1 expression begins to increase, peaks at 48 hours, and then decreases at 72 hours. Furthermore, our experiment in cultured BRL-3A hepatocytes indicates that TMUB1 is highly expressed in the early G2/M phase and gradually decreases as cell mitosis is completed. The above results show that the increasing expression of TMUB1 does not occur earlier than liver regeneration or cell mitosis but occurs simultaneously with liver regeneration or cell mitosis. This finding seems to indicate that high expression of TMUB1 occurs to prevent excessive proliferation of hepatocytes.

Before this study, TMUB1 was known as a shuttle protein between the nucleus and cytoplasm and was mainly localized to the nucleus most of the time. TMUB1 is rapidly exported from the nucleus to cytoplasm during liver regeneration, which is governed by cAMP via CRM-1^1^. We have confirmed that TMUB1 interacts with CAML in the cytoplasm and inhibits BRL-3A hepatocyte proliferation. However, the main purpose of shuttling TMUB1 between the nucleus and cytoplasm is unknown. Potential explanations include transport, a new transcription factor or other functions. We will continue trace the functions of TMUB1 during liver regeneration and hepatocyte proliferation.

This study has some limitations: 1. we could not confirm whether TMUB1 directly associated with CAML or through other proteins. Therefore, we could not determine the mechanisms by whichTMUB1 interfere with binding of CAML to cyclophilin B. *In vitro* protein binding assays (protein pull-down) and exploration of the functional domains of TMUB1 and cyclophilin B will be needed in future experiments; 2. In our experiments of rat partial hepatectomy, we first used the TMUB1 antibodies from Santa Cruz Biotechnology Inc. which only can interact with long isoform (27 kDa, Fig. 1A). However, both long isoform and intermediate isoform (24 kDa) were arrested when we changed these antibodies into that from Abcam Inc. (Fig. 1C,D).

In conclusion, our study first reveals that TMUB1 binds to CAML and disturbs the interaction between CAML and cyclophilin B. Through this mechanism, TMUB1 decreases [Ca2+]i and inhibits BRL-3A hepatocyte proliferation.

## Materials and Methods

### Cell culture

Normal BRL-3 A rat hepatocytes (Cell Bank of Academia Sinica, Shanghai, China) were cultured in high-glucose Dulbecco’s Modified Eagle’s Medium (DMEM) supplemented with 10% fetal bovine serum (Invitrogen Life Technologies, Carlsbad, CA, USA), 2 mM L-glutamine, 0.2 mg/ml streptomycin, and 100 U/ml penicillin at 37 °C with 5% CO2.

### Primary cell culture

Liver tissues were harvested at 0, 12, 24, 48 and 72 hours after liver resection and washed three times with D-Hank’s solution. Tissues were cut into the size of 2~3 millimeter and fully lysed at 37 °C until the tissues were changed into cell suspension. Centrifugating suspension for 5 minutes at 500–1000 rpm and discarding the supernatant. Finally, cells were cultured in high-glucose Dulbecco’s Modified Eagle’s Medium (DMEM) supplemented with 10% fetal bovine serum (Invitrogen Life Technologies, Carlsbad, CA, USA), 2 mM L-glutamine, 0.2 mg/ml streptomycin, and 100 U/ml penicillin at 37 °C with 5% CO2.

### Rat hepatectomy

Experiments were performed on adult male rats (180–200 g). Sprague-Dawleyrats were purchased from the animal experiment center of the Third Affiliated Hospital of the Third Military Medical University. Liver resection was performed at the same time of day, with approximately 70% of liver mass removed. The rats in the sham-operated group (control group) were subjected tolaparotomy under the same conditions. The rats were sacrificed at 0, 12, 24, 48 and 72 hours after liver resection. All methods were carried out in accordance with guidelines and regulations of Chinese Academy of Medical Sciences’s Institute of Laboratory Animal Sciences (ILAS) and all experimental protocols were approved by the Laboratory Animal Welfare and Ethics Committee of the Third Military Medical University (Supplementary: Ethical Statement).

### Bioinformatics productionof the functional domains of Tmub1

According to available rat Tmub1 gene information (GenBank NC_005103.2; http://www.ncbi.nlm.nih.gov/genbank/) and rat Tmub1 protein information (http://www.ncbi.nlm.nih.gov/protein/AAH00936.1), Expert Protein Analysis System (ExPASy) tools (http://expasy.org/tools/) were used to predict the potential functional domains of Tmub1protein. The instability index must be below 40. Another online tool, TMpred (http://www.ch.embnet.org/software/TMPRED_form.html) was used to analyze the transmembrane, hydrophobic and hydrophilic domains of Tmub1.

### Construction of the rat Tmub1 overexpression plasmid and thestructural domain of the Tmub1mutantplasmids

According to available bioinformatics prediction (GenBank NC_005103.2; http://www.ncbi.nlm.nih.gov/genbank/), the cDNA for rat TMUB1 predicts a 738 bp gene encoding a 245-aminoacid protein that contains one ubiquitin-like domain (102–175) encoded by 304–525 bp and two C-terminal hydrophobic domains, including TM1 (194–214) encoded by 580–642 bp and TM2 (219–239) encoded by 655–717 bp (Supplementary: Gene sequence). Full-length Tmub1 and its mutant constructs were obtained throughstandard PCR-based mutagenesis protocols (QuikChangeSite-Directed Mutagenesis Kit; Stratagene). Every pcDNA was Flag-tagged. The Flag encoding sequence was ATGGATTACAAGGATGACGACGATAAG. The expression plasmid pcDNA-FLAG-Tmubl contained the full-length gene (amino acids 1–245). The expression plasmid pcDNA-FLAG-△TM1 contained deletions of the C-terminal hydrophobic domain (amino acids 194–214). The expression plasmid pcDNA-FLAG-△TM2 contained deletions of the second C-terminal hydrophobic domain (amino acids 219–239). The expression plasmid pcDNA-FLAG-△U1 contained deletions of the first section of the UBL domain (amino acids 102–138). The expression plasmid pcDNA-FLAG-△U2 contained deletions of the second section of the UBL domain (amino acids 139–175).

### Bacteria culture and plasmid extraction

Competent bacteria and encapsulated plasmid DNA were obtained from Invitrogen. A total of 50 µl bacteria solution, 20 ml LB medium and 40 µl ampicillin sodium were placed in aconical flask and shakenat 37 °C for16 hours. After the LB medium becameturbid, a 10-ml suspension was centrifuged under10,000 rpm for 4 min. Thesupernatant was then discarded, and theplasmid was extracted with an EndoFree Plasmid Midi Kit (Cwbiotech, Beijing, China). The concentration of the plasmid was tested with aspectrophotometer (NV3000C, Thermo Fisher Scientific Inc., Waltham, MA USA).

### Cell transfection and transfection efficiency detection

BRL-3 A rat hepatocytes were cultured in high-glucose DMEM supplemented with 10% fetal bovine serum at 37 °C with 5% CO2. When the hepatocytes reached 80–90% confluence, they were passaged and placed in a 100-mm culture dish. After 24 hours, when the cells had reached 40–60% confluence, they were passaged and placed in DMEM without penicillin-streptomycin. A total of 72 µl LipoFiter^TM^ was mixed with 928 ml DMEM in a 100-mm culture dish and was not disturbed for 5 min. The final concentration of the plasmid was 24 µg DNA/1 ml DMEM. The two above-mentioned solutions were mixed, left undisturbed for 20 min and added to DMEM in a 100-mm culture dish with a final volume of 12 ml. The cells were cultured at 37 °C with 5% CO2 for 4–6 hours and were then changed from DMEM to high-glucose DMEM supplemented with 10% fetal bovine serum. After 24 hours, the cells were collected, and protein was extracted for Western blot analysis with an anti-Flag antibody (Supplementary Figs 1, 2). PCR was also used to test whether the transfection was successful. The sense and antisense primers for pcDNA-FLAG-Tmub1 and its mutant amplification are as follows: 5′-AAGGATGACGACGATAAGAT-3′ and 5′-GAGGTGTTGATGCTGTGA-3′, respectively (Supplementary Fig. 3).

### RNA interference

Tmub1 RNA interference has been tested many times by our research team in previous studies^23,27^. Based on these previous results, we determined that Tmub1 siRNA496, which was designed and synthesized by Dharmacon/Thermo Fisher Scientific, was the best RNA interference strategy (Figs 4A and 7H). The sequence of siRNA496 is as follows: GGUUCGACUCAUCUACCAATT. A total of 46 µl siRNA solution was added to 954 µl DMEM and then mixed with 72 µl LipoFiter^TM^ and 928 ml DMEM in a 100-mm culture dish. The remaining procedures were the same as those used for plasmid transfection.

### Real-time quantitative RT-PCR analysis (Q-PCR)

RNA was extracted using TRIzol reagent (I Invitrogen Life Technologies, Carlsbad, CA, USA); the extracted RNA was collected and purified by a Qiagen RNeasy Kit (Qiagen, Germany). Prepared RNA was used for reverse transcription with a QiagenQuantiTect Reverse Transcription Kit according to the manufacturer’s instructions. PCR reactions were carried out with a Brilliant SYBR Green qPCR Master Mix Kit (Qiagen, German). The sequences of primers and the internal reference β-actin were designed, synthesized and analyzed by BioSpaceBio-tech Inc. (Chongqing, China). The sense and antisense primers for CAML amplification are as follows: 5′-CATCAACCGGATCATGGGCTT-3′ and 5′-TCGCTTTGAAACGGAA GGAAC-3′, respectively. The sense and antisense primers for β-actin amplification are as follows: 5′-ACCCTGAAGTACCCCATCGAG-3′ and 5′-ACATGATCTGGGT CATCTTCTCG-3′, respectively.

### Co-Immunoprecipitation

Transfected BRL-3 A cells were collected, harvested and washed in moderate celllysis buffer (20 mMTris-HCl, pH 7.5; 500 mMNaCl; 2% Triton X-100) containing protease inhibitor. The cells were fully lysed by 30 min at 0–4 °C. Thesamples were centrifuged at 13,000 rpm for 10 min at 4 °C to obtainsupernatant for future experiments. A total of 30 µl supernatant was mixed with 7–8 µl 5 × SDS-PAGE loading buffer to serveasa positive control. Magnetic beads (Surebeads Magnetic Beads, Bio-Rad, Shanghai, China) were thoroughly resuspended, and 50–60 µl beadswere transferred to 2-ml tubes. The beads were magnetized, and the supernatant was discarded. The beads were then washed three times with 1,000 µl PBS-T. A total of 1–10 µg antibody was added toafinal volume of 200 µl, and the beads were resuspended (anti-IgG antibody for negative control). The mixture was rotated for 30 min at room temperature. The beads were magnetized, the supernatant was discarded, and the beads were washedthree times with 1,000 µl PBS-T. Next, 500 µl protein lysate (containing 500 µg/mg protein) was rotatedovernight at 4 °C. The beads were magnetized, and thesupernatant was discarded. Then, thebeads were washedwith PBS-T. The beads were magnetized, and residual buffer was aspirated from the tubes. A total of 30 µl 1 × SDS-PAGE loading bufferwas added to the beadsand incubated for 10 min at 70 °C. The supernatant was taken for Western blot analysis.

### Measurement of [Ca2+]i levels

The cells were grown and detached with 0.25% trypsin into a cell suspension, washed three times with D-Hank’s solution, and incubated with 2 μM Fura-2/AM for 30 min at 37 °C in a 5% (v/v) CO2 incubator. Then, the cells were centrifuged to discard Fura-2/AM-containing DMEM and washed three times with 37 °C D-Hank’s solution before being transferred into a new culture dish especially designed for inverted fluorescence microscopy. The cells were then viewed under a fluorescence microscope (Nikon Eclipse Ti, Tokyo, Japan); the excitation wavelength was between 340 and 380 nm. Images were obtained from five randomly selected fields, and at least 10 cells were observed per visual field. The results were analyzed using Nis-Elements software (Nikon Eclipse Ti, Tokyo, Japan) and presented as the mean ± standard deviation (SD).

### Measurement of calcium concentration

Primary cells were incubated with 5 μM Fluo-3 AM solution (Thermo Fisher Scientific Inc., Waltham, MA USA) for 60 min at 37 °C in a 5% (v/v) CO2 incubator and washed with PBS solution three times. Moreover, cells were incubated for another 20–30 min at 37 °C in a 5% (v/v) CO2 incubator to comfirm that the Fluo-3 AM changed into Fluo-3 in the cells. Calcium concentration was test by flow cytometer and analyzed by CXP software.

### Cell cycle synchronization

The BRL-3A cell cycle was synchronized at the G0/G1 boundary in serum-freemedium, in the G2/M0 phase by the nocodazole method and in the G1/S phase by the double thymidine method. Generally, BRL-3A cells were cultured in the absence of serum, harvested after 72 hours andreleased in DMEM supplemented with 10% fetal bovine serum. The time point at which BRL-3A cells received serum after starvation was considered time 0. The double thymidine method to arrest the cells at the G1/S boundary is as follows: when BRL-3A cells reached 30% confluence, themedium was changed to DMEM supplemented with 10% fetal bovine serum containing a final concentration of 2 mM thymidine. After 18 hours, thethymidine medium was changed to DMEM supplemented with 10% fetal bovine serum, the cells were cultured for 6 hours, and the medium was then changed to DMEM containing a final concentration of 2 mM thymidine. The BRL-3A cells were cultured in thymidine, harvested after 12 hours and released in DMEM supplemented with 10% fetal bovine serum. The nocodazole method to arrest the cells at the G2/M0 boundary is as follows: nocodazole was added to DMEM supplemented with 10% fetal bovine serumat a 330 nM final concentration. The BRL-3Acells were cultured in nocodazole, harvested after18 hours andreleased in DMEMsupplemented with 10% fetal bovine serum. Cell cycle synchronization was tested by flow cytometry (Supplementary Fig. 4A–O).

### Western blot analysi

We collected and washed the cells with PBS and then lysed the cells inthe following solution system on ice: RIPAlysate, protease inhibitor and phosphatase inhibitor (98:1:1). Protein samples were separated by SDS-PAGE and transferred to a nitrocellulose membrane. The membrane was incubated with rabbit anti-mouse primary antibodies and goat anti-rabbit secondary antibodies (Santa Cruz Biotechnology Inc., Santa Cruz, CA, USA and Abcam Inc., Cambrige, British) in 5% non-fat dry milk buffer for 1 hour. Protein bands were imaged by an Odyssey Two Infrared Imaging System (LI-COR, Lincoln, NE, USA) and analyzed by Quantity One software (Bio-Rad).

### Immunofluorescence and laser confocal scanning

For immunofluorescence microscopy, the cells were grown on coverslips, fixed with 4% paraformaldehyde and incubated overnight withanti-TMUB1 and anti-CAML antibodies. Proteins were visualized by incubation with goatanti-rabbit antibodies conjugated to Alexa Fluor 488 (Invitrogen, Carlsbad, CA, USA). Finally, the coverslips were incubated with 4,6-diamidino-2-phenylindole (DAPI, Sigma-Aldrich) for 10 min and visualized under a fluorescence microscopeand laser scanning confocal microscope.

### EDU assays and CCK-8 assays

Transfected cells were cultured in 96-well plates and tested with a Click-iT Edu Alexa Fluor Test kit. The cells were inoculated in 96-well plates with 1000 cells/well following transfection with pcDNA-TMUB1-Flag, pcDNA-ΔTM1-Flag and pcDNA-control. A flash, full wavelength scanning, multifunction reading instrument was used to count the cells at 0, 24, 48 and 72 hours after WST-8 was added to the wells.

### Statistical analysis and image processing

Data were analyzed with SPSS software, version 16.0, and all reported values represent the mean and SD of at least three independent experiments. Enumeration data were evaluated with chi-square test. Least-Significant Difference test was used to determine differences among these independent subgroups. A P-value < 0.05 was considered statistically significant. Gel images were treated by Image Lab version 5.1. The gray value test was treated by ImageJ. Histograms and Immunofluorescence diagrams were treated by GraphPad Prism 5. Image synthesis was treated by Microsoft powerpoint 2007.

## Electronic supplementary material

Supplementary Information
