# Supplementary material for: TMUB1 Inhibits BRL-3A Hepatocyte Proliferation by Interfering with the Binding of CAML to Cyclophilin B through its TM1 Hydrophobic Domain
Source: Sci Rep. 2018 Jul 2;8:9917. doi: 10.1038/s41598-018-28339-4 (PMC6028644; doi:10.1038/s41598-018-28339-4)
Supplement: Supplementary file 1 — Supplementary Information [file 41598_2018_28339_MOESM1_ESM.pdf]

TMUB1 Inhibits BRL-3A Hepatocyte Proliferation by Interfering with the Binding of CAML to Cyclophilin B through its TM1 Hydrophobic Domain.

Xiang Lan, Hangwei Fu, Guangyao Li, Wei Zeng, Xia Lin, Yuanxin Zhu, Menggang Liu*, Ping Chen*

Department of Hepatobiliary Surgery, the Third Affiliated Hospital, the Third Military Medical University, Chongqing, China

*Correspondence to: Menggang Liu or Ping Chen, MD, PhD, Department of Hepatobiliary Surgery, the Third Affiliated Hospital, the Third Military Medical University, 10 Changjiang Branch Road, Yuzhong district Chongqing, China.Postal code: 400042 Email: liumenggang1978@163.com (Menggang Liu) or chenpingsyd@126.com (Ping Chen)

Telephone: 0086-023-68757968 Fax: 0086-023-68757963

Financial support:

1.Supported by the Innovation Funds of the Third Affiliated Hospital ofthe Third Military Medical University (No. 50116-2299): The mechanism of TMUB1's interaction with CAML during liver regeneration.

2.Supported by the National Nature Science Foundation of China (No. 81270523): Influence of Tmub1 protein on the ubiquitination-like mechanism of securin in the hepatocyte cycle in liver regeneration.

Gene sequence

Tmubl sequence:

Transmembrane and ubiquitin-like domain-containing protein 1, Rattus norvegicus (245 aa)

MALIEGVGDEVTVLFSVLACLLVLALAWVSTHTTESTDPLPQSSGTTTPAQPSEAMTAIDSIREEAPGAESPSLRH
RGPSAQPEPEAGVTASTPPDSPQEPLLLRLKFLNDSEQVARAWPQDTIGSLKRTQFPGREQQVRLIYQGQLLGD
DTQTLGSLHLPPNCVLHCHVSTRVGPPHPPCPPGSEPGPSGLEIGSLLLPLLLLLLLLWYCQIQYRPFFPLTATLGL
AGFTLLLSLLAFAMYRP

TM1 TM2 UL

TMUB1 CDS sequence:

Atggccttgattgaaggcgtaggggatgaggtgactgtcctttttcggtgcttgcctgccttctggtgctggccctcgcctgggtctcaacaca
tacgactgagagtacagatcccctaccacagtcgtcagggaccacaacaccagcacagcccagtgaagccatgacagccattgatagcatc
agagaggaggccccaggagctgagagtcccagcctgaggcacagaggtccatctgcacagccagagcctgaggcaggggtcacagcatc
aacacctccagactctccacaggaacccttactgctacggttgaaatttctcaatgactctgaacaggtggccagggcctggcctcaggaca
ccattggctccttgaaaagaacccagtttccaggccgggaacagcaggttcgactcatctaccaaggccaactgctaggagacgacaccca
gacactgggcagtctccaccttccccccaactgcgttctccactgccacgtgtccacaagagtcggtcccccgcatcctccctgcccaccggg
gtcagagcccggcccctccgggctggaaatcggcagccttctgttgcccctgctgcttctgctgctgctcctgctctggtactgccagatccagt
accggcccttctttcccctgaccgctaccttgggcctggccggcttcaccctgctcctcagtctcctggcctttgccatgtatcgcccgtag

TM1 (580-642 bp) TM2 (655-717 bp) UL (304-525 bp)

Flag tag coded sequence:

ATGGATTACAAGGATGACGACGATAAG

Synthetic sequences

Flag-Tmubl

ATGGATTACAAGGATGACGACGATAAGatggccttgattgaaggcgtaggggatgaggtgactgtcctttttcggtgcttgcctg
ccttctggtgctggccctcgcctgggtctcaacacatacgactgagagtacagatcccctaccacagtcgtcagggaccacaacaccagcac
agcccagtgaagccatgacagccattgatagcatcagagaggaggccccaggagctgagagtcccagcctgaggcacagaggtccatctg
cacagccagagcctgaggcaggggtcacagcatcaacacctccagactctccacaggaacccttactgctacggttgaaatttctcaatgac
tctgaacaggtggccagggcctggcctcaggacaccattggctccttgaaaagaacccagtttccaggccgggaacagcaggttcgactcat
ctaccaaggccaactgctaggagacgacacccagacactgggcagtctccaccttccccccaactgcgttctccactgccacgtgtccacaa
gagtcggtcccccgcatcctccctgcccaccggggtcagagcccggcccctccgggctggaaatcggcagccttctgttgcccctgctgcttct
gctgctgctcctgctctggtactgccagatccagtaccggcccttctttcccctgaccgctaccttgggcctggccggcttcaccctgctcctca
gtctcctggcctttgccatgtatcgcccgtag

1. Flag-△TM1：knockout 580—642 bp

ATGGATTACAAGGATGACGACGATAAGatggccttgattgaaggcgtaggggatgaggtgactgtcctttttcggtgcttgcctgc
cttctggtgctggccctcgcctgggtctcaacacatacgactgagagtacagatcccctaccacagtcgtcagggaccacaacaccagcaca
gcccagtgaagccatgacagccattgatagcatcagagaggaggccccaggagctgagagtcccagcctgaggcacagaggtccatctgc
acagccagagcctgaggcaggggtcacagcatcaacacctccagactctccacaggaacccttactgctacggttgaaatttctcaatgact
ctgaacaggtggccagggcctggcctcaggacaccattggctccttgaaaagaacccagtttccaggccgggaacagcaggttcgactcat
ctaccaaggccaactgctaggagacgacacccagacactgggcagtctccaccttccccccaactgcgttctccactgccacgtgtccacaa
gagtcggtcccccgcatcctccctgcccaccggggtcagagcccggcccctccgggctggaacagtaccggcccttctttcccctgaccgcta
ccttgggcctggccggcttcaccctgctcctcagtctcctggcctttgccatgtatcgcccgtag

2. Flag-△TM2：knockout 655—717 bp

ATGGATTACAAGGATGACGACGATAAGatggccttgattgaaggcgtaggggatgaggtgactgtcctttttcggtgcttgcctgc
cttctggtgctggccctcgcctgggtctcaacacatacgactgagagtacagatcccctaccacagtcgtcagggaccacaacaccagcaca
gcccagtgaagccatgacagccattgatagcatcagagaggaggccccaggagctgagagtcccagcctgaggcacagaggtccatctgc
acagccagagcctgaggcaggggtcacagcatcaacacctccagactctccacaggaacccttactgctacggttgaaatttctcaatgact
ctgaacaggtggccagggcctggcctcaggacaccattggctccttgaaaagaacccagtttccaggccgggaacagcaggttcgactcat
ctaccaaggccaactgctaggagacgacacccagacactgggcagtctccaccttccccccaactgcgttctccactgccacgtgtccacaa

gagtcggtcccccgcatcctccctgcccaccggggtcagagcccggcccctccgggctggaa atcggcagccttctgttgcccctgctgcttct

gctgctgctcctgctctggtactgccagatccagtaccggccctttgccatgtatcgcccgtag

3. **Flag-△UL1：knockout 304-453 bp**

ATGGATTACAAGGATGACGACGATAAGatggccttgattgaaggcgtaggggatgaggtgactgtcctttttcggtgcttgcctg

ccttctggtgctggccctcgcctgggtctcaacacatacgactgagagtacagatcccctaccacagtcgtcagggaccacaacaccagcac

agcccagtgaagccatgacagccattgatagcatcagagaggaggccccaggagctgagagtcccagcctgaggcacagaggtccatctg

cacagccagagcctgaggcaggggtcacagcatcaacacctccagactctccacaggaacccacccagacactgggcagtctccaccttcc

ccccaactgcgttctccactgccacgtgtccacaagagtcggtcccccgcatcctccctgcccaccggggtcagagcccggcccctccgggct

ggaaatcggcagccttctgttgcccctgctgcttctgctgctgctcctgctctggtactg ccagatccagtaccggcccttctttcccctgaccgc

taccttgggcctggccggcttcaccctgctcctcagtctcctggcctttgccatgtatcgcccgtag

4. Flag-△UL2：knockout 376-525 bp

ATGGATTACAAGGATGACGACGATAAGatggccttgattgaaggcgtaggggatgaggtgactgtcctttttcggtgcttgcctg

ccttctggtgctggccctcgcctgggtctcaacacatacgactgagagtacagatcccctaccacagtcgtcagggaccacaacaccagcac

agcccagtgaagccatgacagccattgatagcatcagagaggaggccccaggagctgagagtcccagcctgaggcacagaggtccatctg

cacagccagagcctgaggcaggggtcacagcatcaacacctccagactctccacaggaacccttactgctacggttgaaatttctcaatgac

tctgaacaggtggccagggcctggcctcaggacaccattggccccccgcatcctccctgcccaccggggtcagagcccggcccctccgggct

ggaaatcggcagccttctgttgcccctgctgcttctgctgctgctcctgctctggtactgccagatccagtaccggcccttctttcccctgaccgc

taccttgggcctggccggcttcaccctgctcctcagtctcctggcctttgccatgtatcgcccgtag

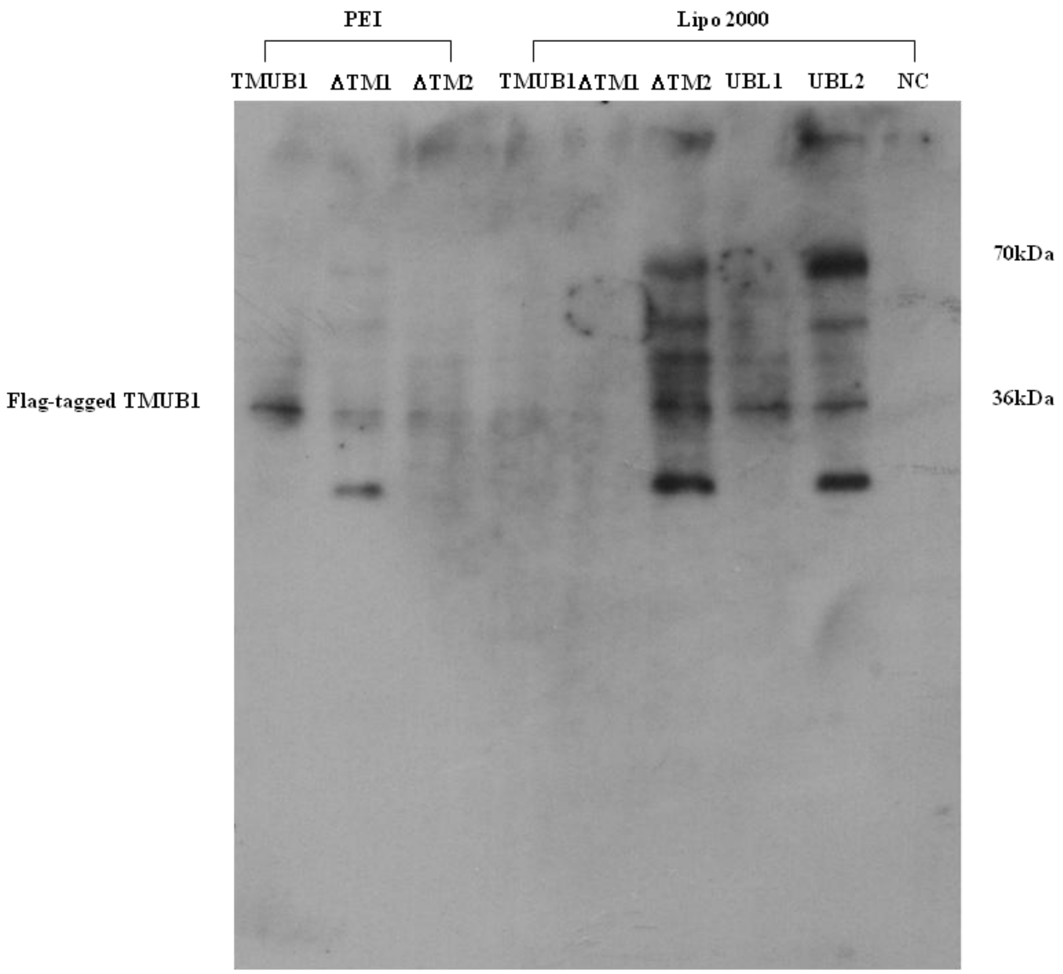

Fig 1. The first plasmid transfection with BRL-3A hepatocytes. PEI and Lipo 2000 was tested repectively. BRL-3A rat hepatocytes were cultured in high-glucose DMEMsupplemented with 10% fetal bovine serum at 37°C with 5% CO2. When the hepatocytesreached 80-90% confluence, they were passagedand placed in a 100-mm culture dish.After 24 hours, when the cells had reached 40-60% confluence, they were passaged and placed in DMEM without penicillin-streptomycin. A total of 21 μl PEI or 72ulLipoFiterTM was mixed with 979ml DMEM (PEI) or 928ml DMEM (Lipo 2000) in a 100-mm culture dish and was not disturbed for 5 min. The final concentration of the plasmid was 7-8ug (PEI) or 24 μg (Lipo 2000) DNA/1ml DMEM. The two abovementioned solutions were mixed, left undisturbedfor 20 min and added to DMEM in a 100-mm culture dish with a final volume of 12 ml. The cells were cultured at 37°C with 5% CO2 for 7 hours and were then changed from DMEM to high-glucose DMEM supplemented with 10% fetal bovine serum. After 24 hours, the cells were collected, and protein was extracted for Western blot analysis with an anti-Flag antibody.

In this transfection experiment, we can see flag-tagged TMUB1 was tested in both kinds of transfection solution but with lower transfection efficiency. Hepatocytes suffered from extensive death. We thought it was due to long time incubation with transfection solution. So we operated a scond transfection experiments and decreased the incubation time to 4-6 hours.

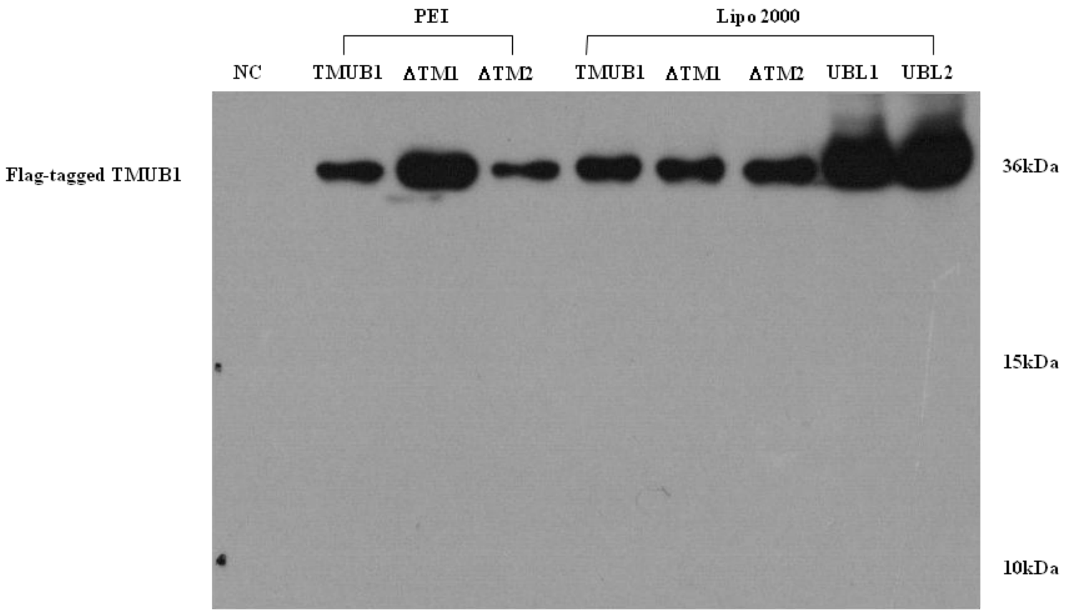

Fig 2. The second plasmid transfection with BRL-3A hepatocytes. PEI and Lipo 2000 was tested repectively. BRL-3A rat hepatocytes were cultured in high-glucose DMEMsupplemented with 10% fetal bovine serum at 37˚C with 5% CO2. When the hepatocytesreached 80-90% confluence, they were passagedand placed in a 100-mm culture dish.After 24 hours, when the cells had reached 40-60% confluence, they were passaged and placed in DMEM without penicillin-streptomycin. A total of 21 µl PEI or 72ulLipoFiterTM was mixed with 979ml DMEM (PEI) or 928ml DMEM (Lipo 2000) in a 100-mm culture dish and was not disturbed for 5 min. The final concentration of the plasmid was 7-8ug (PEI) or 24µg (Lipo 2000) DNA/1ml DMEM. The two abovementioned solutions were mixed, left undisturbedfor 20 min and added to DMEM in a 100-mm culture dish with a final volume of 12 ml. The cells were cultured at 37˚C with 5% CO2 for 4-6 hours and were then changed from DMEM to high-glucose DMEM supplemented with 10% fetal bovine serum. After 24 hours, the cells were collected, and protein was extracted for Western blot analysis with an anti-Flag antibody.

In this transfection experiment, we can see flag-tagged TMUB1 was tested in both kinds of transfection solution with satified transfection efficiency. Hepatocytes suffered from extensive death. We thought it was due to long time incubation with transfection solution. So we operated a scond transfection experiments and decreased the incubation time to 4-6 hours.

So we choosed lipo 2000 as the transfection solution and incubation time was 4-6 hours.

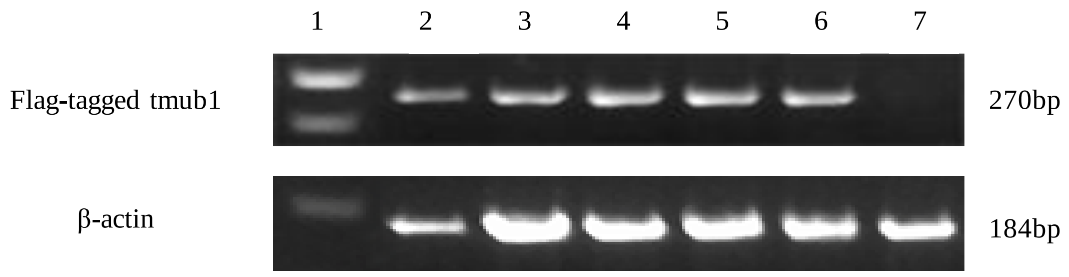

1. DNA marker
2. pcDNA-FLAG-Tmubl
3. pcDNA-FLAG-∆Tm2
4. pcDNA-FLAG-∆U1
5. pcDNA-FLAG-∆U2
6. pcDNA-FLAG

Fig3. PCR test for hypatocytes plasmid transfection. The sense primers are located in the region of Flag and the anti-sense primers are licated in the region of Tmub1. The sense and antisense primers for pcDNA-FLAG-Tmub1 and its mutant amplification are as follows: 5'-AAGGATGACGACGATAAGAT-3' and 5'-GAGGTGTTGATGCTGTGA-3', respectively.

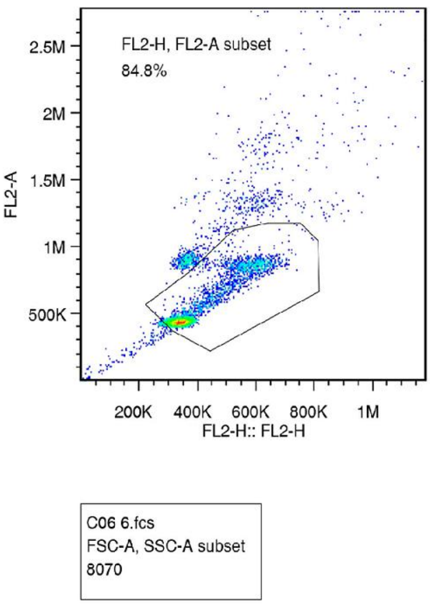

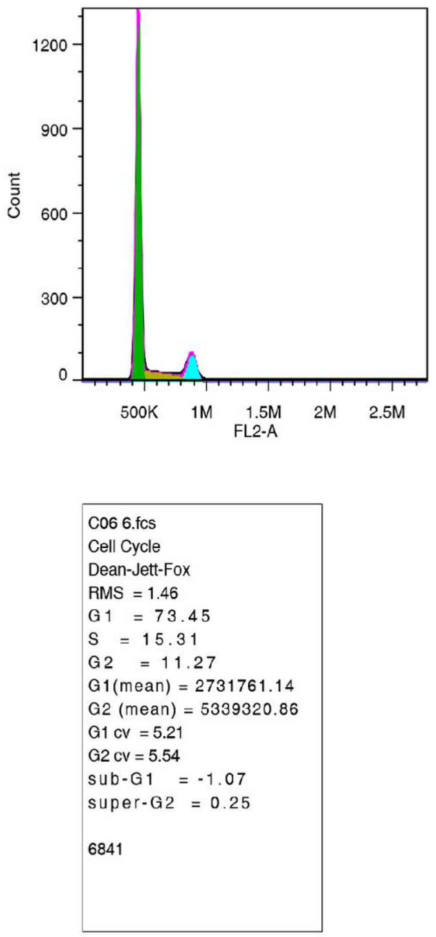

Fig4A. Cell cycle tested: G0/G1 phase 0h

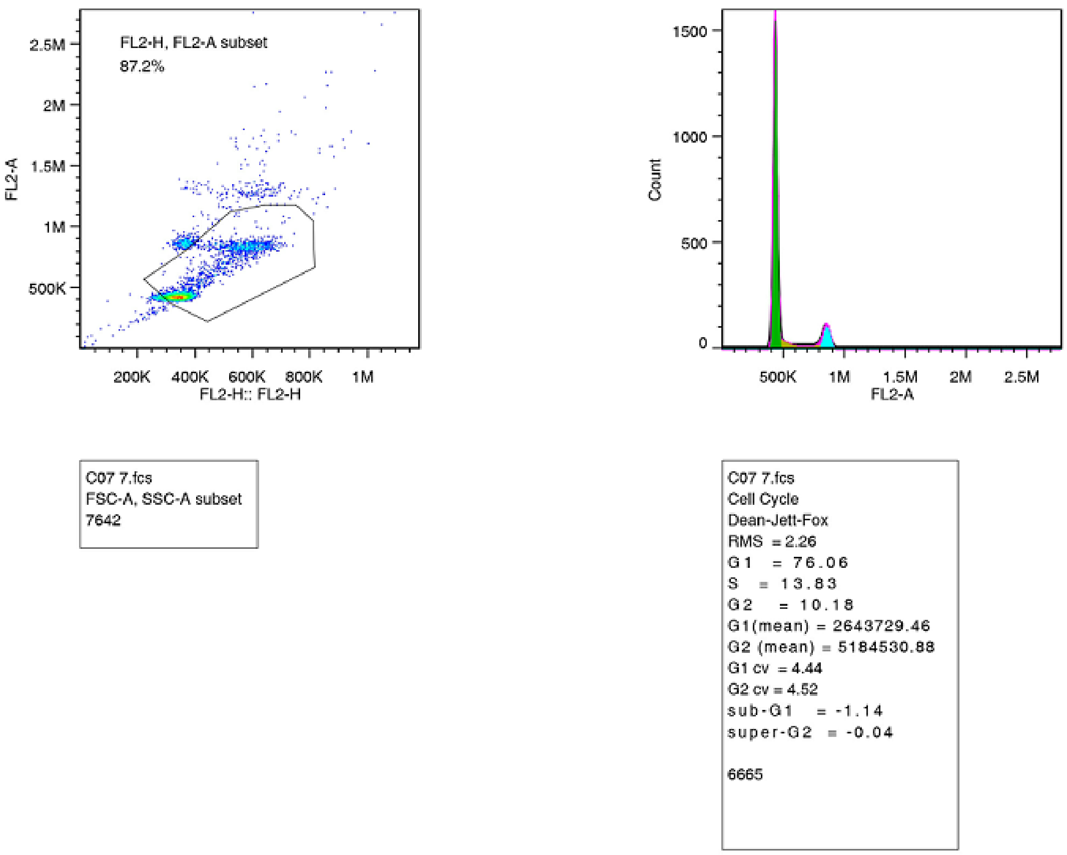

Fig4B. Cell cycle tested: G0/G1 phase 2h

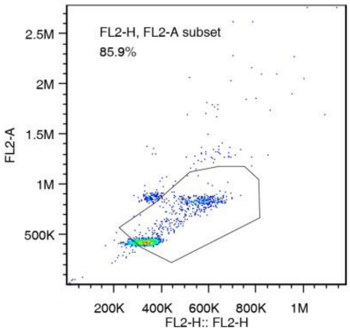

C08 8.fcs
FSC-A, SSC-A subset
3251

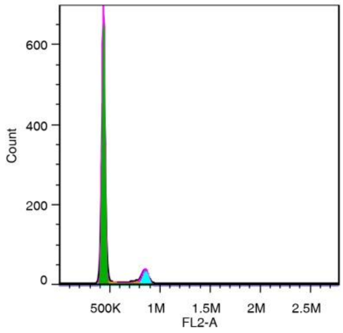

C08 8.fcs
Cell Cycle
Dean-Jett-Fox
RMS = 1.13
G1 = 80.79
S = 10.71
G2 = 8.4
G1(mean) = 2651772.87
G2 (mean) = 5185776.67
G1 cv = 4.56
G2 cv = 4.78
sub-G1 = -0.56
super-G2 = -0.2

2794

Fig4C. Cell cycle tested: G0/G1 phase 4h

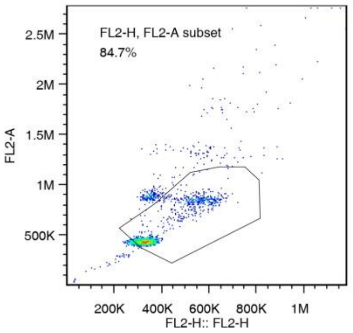

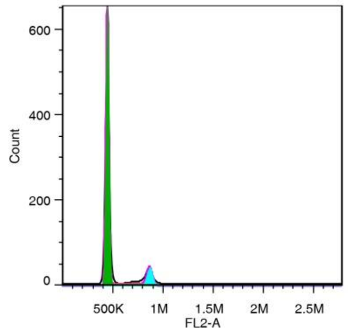

C09 9.fcs
FSC-A, SSC-A subset
3440

C09 9.fcs
Cell Cycle
Dean-Jett-Fox
RMS = 1.06
G1 = 82.83
S = 7.29
G2 = 9.54
G1(mean) = 2708180.14
G2 (mean) = 5246335.81
G1 cv = 4.82
G2 cv = 4.61
sub-G1 = -0.21
super-G2 = 0.73

2914

Fig4D. Cell cycle tested: G0/G1 phase 6h

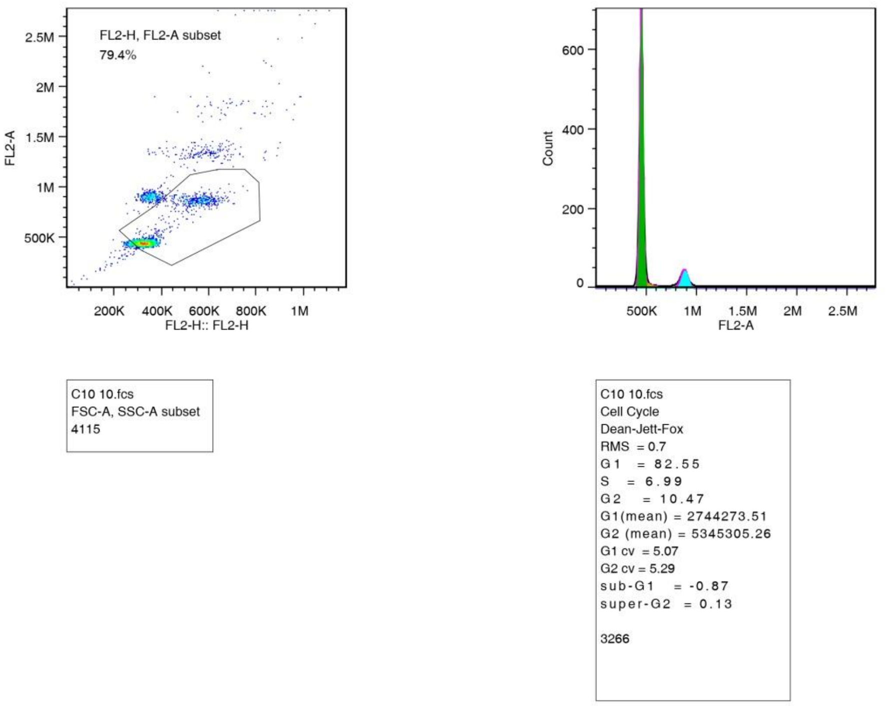

Fig4E. Cell cycle tested: G0/G1 phase 8h

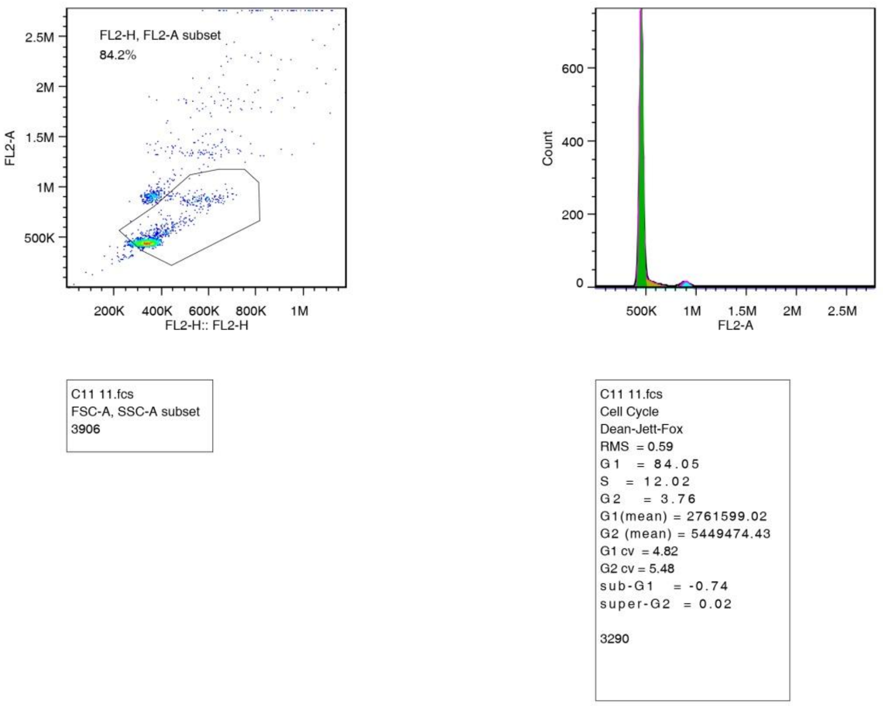

Fig4F. Cell cycle tested: G0/G1 phase 10h

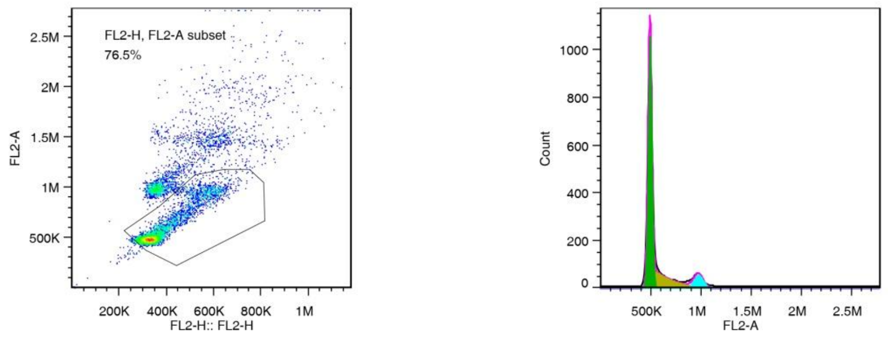

C12 12.fcs
FSC-A, SSC-A subset
9655

C12 12.fcs
Cell Cycle
Dean-Jett-Fox
RMS = 1.52
G1 = 67.79
S = 22.69
G2 = 8.62
G1(mean) = 2981514.39
G2 (mean) = 5898221.95
G1 cv = 5.67
G2 cv = 6.89
sub-G1 = -0.85
super-G2 = 0.13

7390

Fig4G. Cell cycle tested: G0/G1 phase 12h

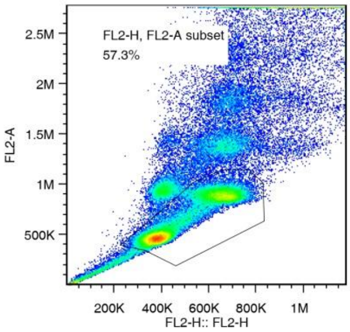

A01 1.fcs
FSC-A, SSC-A subset
87522

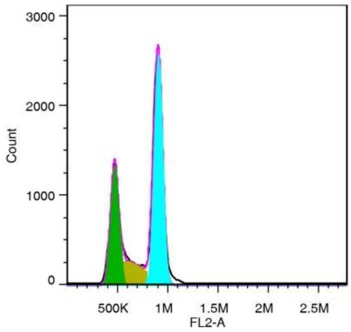

A01 1.fcs
Cell Cycle
Dean-Jett-Fox
RMS = 8.38
G1 = 14.51
S = 5.15
G2 = 78.94
G1(mean) = 2864254.45
G2 (mean) = 5475712.71
G1 cv = 10.63
G2 cv = 7.32
sub-G1 = -0.61
super-G2 = 1.04

50122

Fig4H. Cell cycle tested: G2/M phase 0h

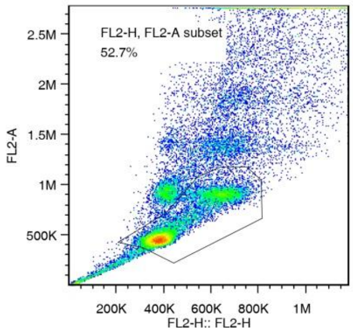

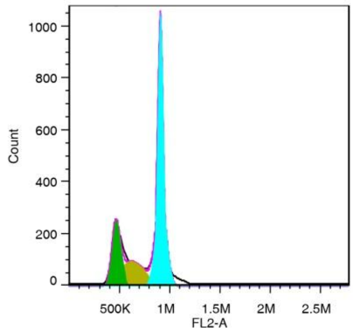

A02 2.fcs
FSC-A, SSC-A subset
28528

A02 2.fcs
Cell Cycle
Dean-Jett-Fox
RMS = 4.21
G1 = 10.64
S = 6.75
G2 = 79.32
G1(mean) = 2818657.07
G2 (mean) = 5513306.6
G1 cv = 10.88
G2 cv = 8.14
sub-G1 = -0.95
super-G2 = 0.64

15045

Fig4I. Cell cycle tested: G2/M phase 1h

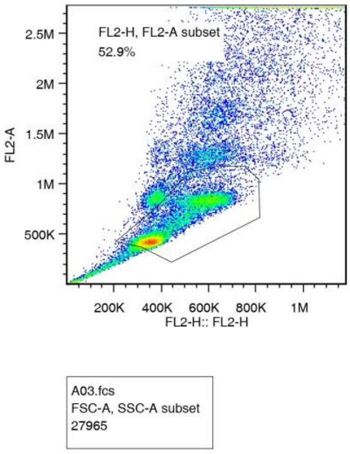

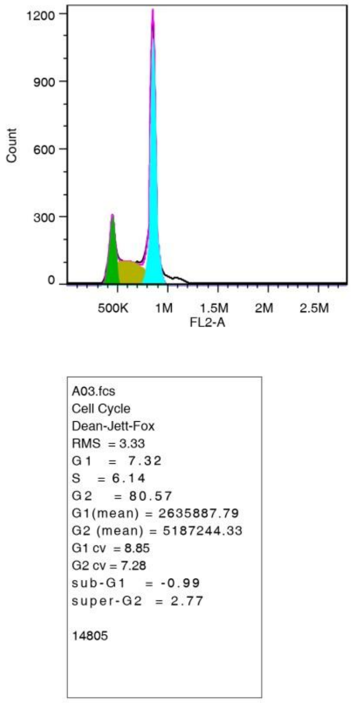

Fig4J. Cell cycle tested: G2/M phase 2h

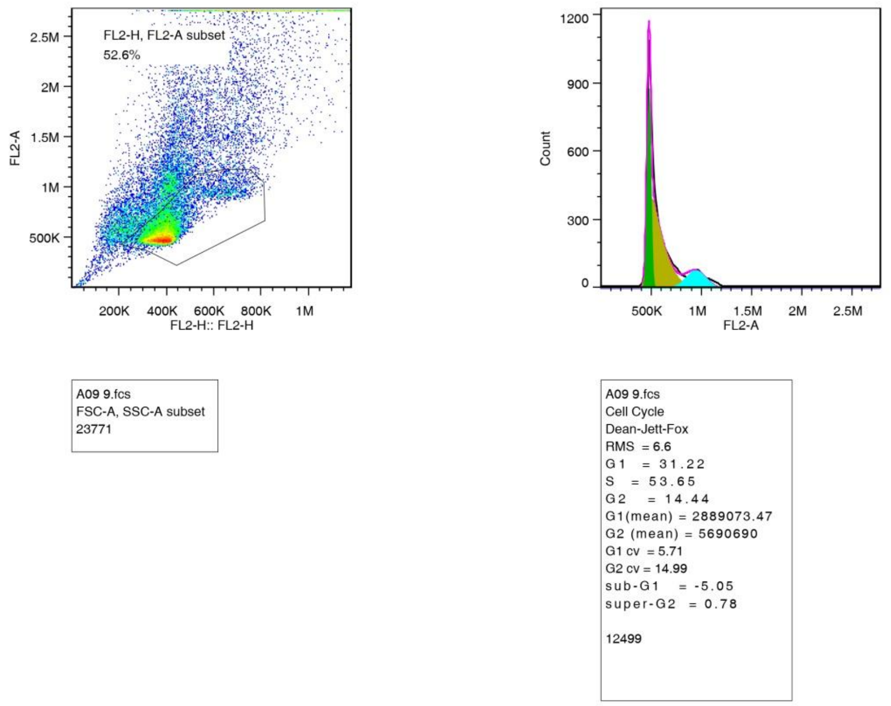

Fig4K. Cell cycle tested: G1/S phase 0h

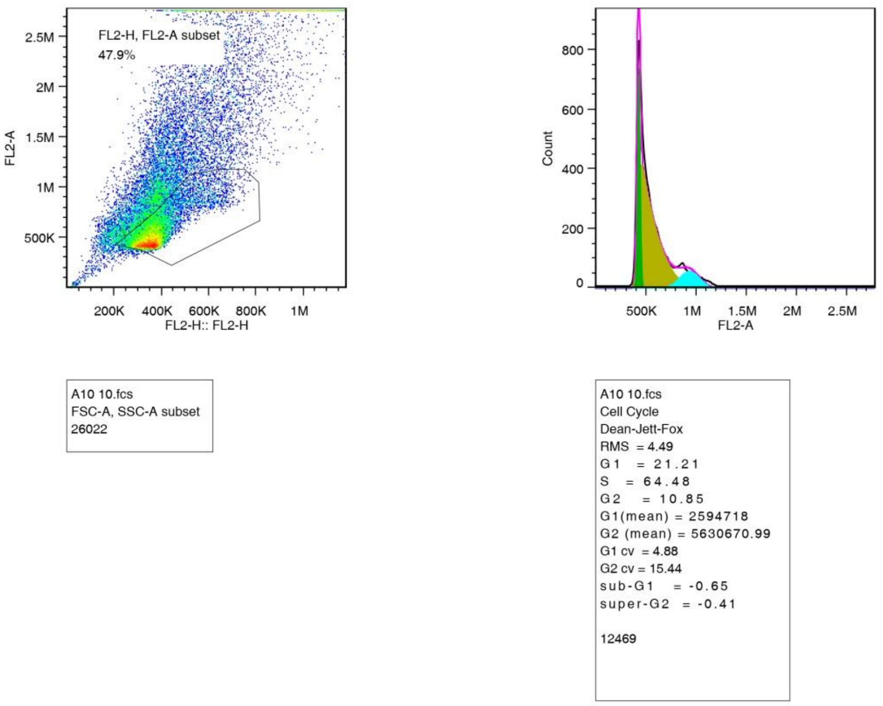

Fig4L. Cell cycle tested: G1/S phase 2h

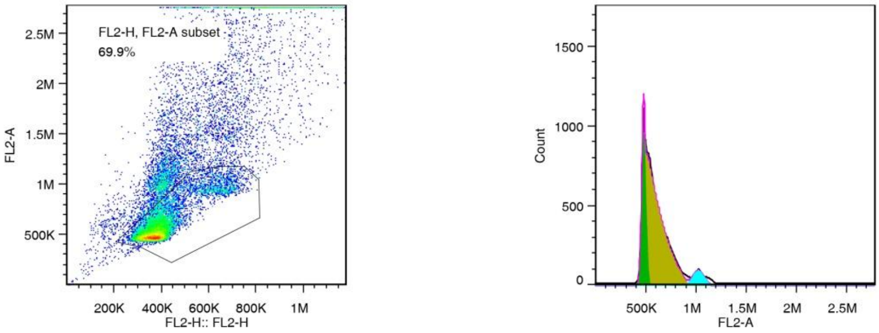

A06 6.fcs
FSC-A, SSC-A subset
20238

A06 6.fcs
Cell Cycle
Dean-Jett-Fox
RMS = 7.2
G1 = 17.11
S = 64.68
G2 = 10.37
G1(mean) = 2899893.39
G2 (mean) = 5720175
G1 cv = 4.95
G2 cv = 12.03
sub-G1 = -4.07
super-G2 = 1.88

14151

Fig4M. Cell cycle tested: G1/S phase 4h

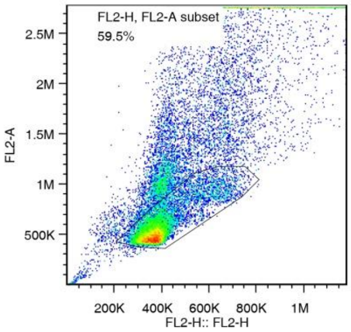

A07 7.fcs
FSC-A, SSC-A subset
22780

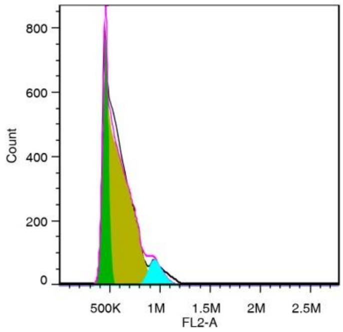

A07 7.fcs
Cell Cycle
Dean-Jett-Fox
RMS = 8.52
G1 = 12.46
S = 61.34
G2 = 10.75
G1(mean) = 2830602
G2 (mean) = 5484292
G1 cv = 9.76
G2 cv = 15.61
sub-G1 = -1.29
super-G2 = 0.8

13558

Fig4N. Cell cycle tested: G1/S phase 6h

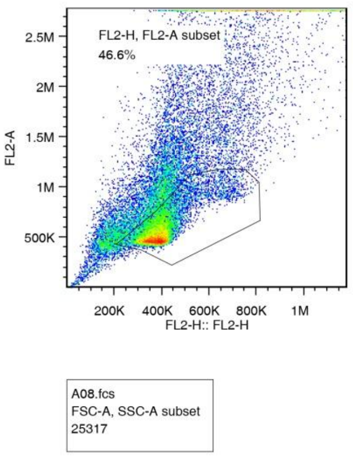

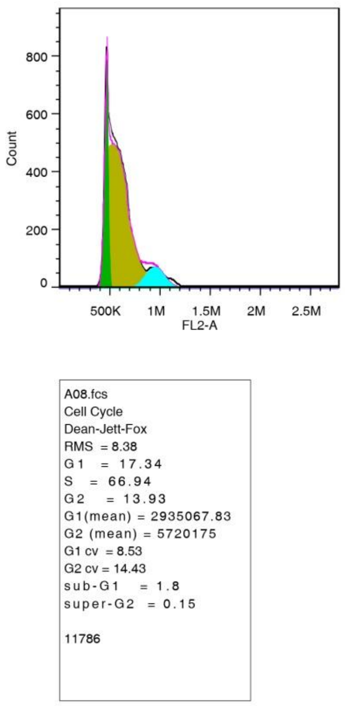

Fig4O. Cell cycle tested: G1/S phase 8h

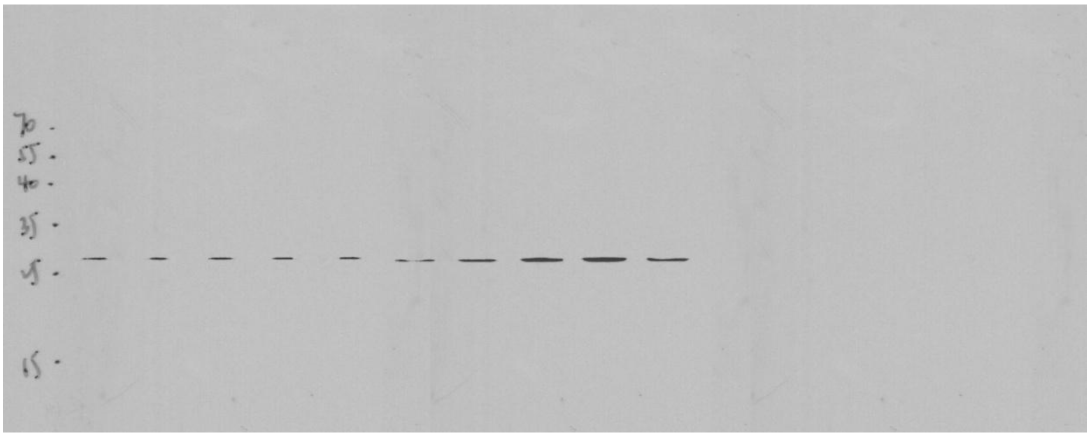

Fig5A. Original gel for TMUB1 expression in sham-operated and operated group

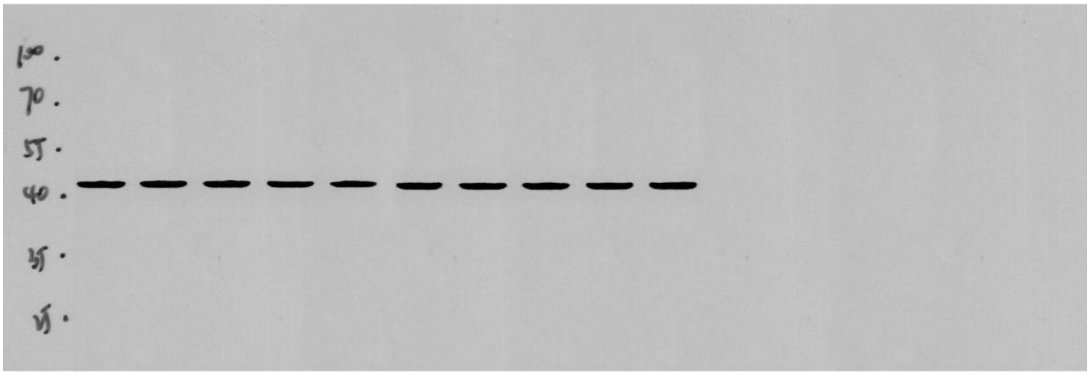

Fig5B. Original gel forβ -actin expression in sham-operated andoperated group

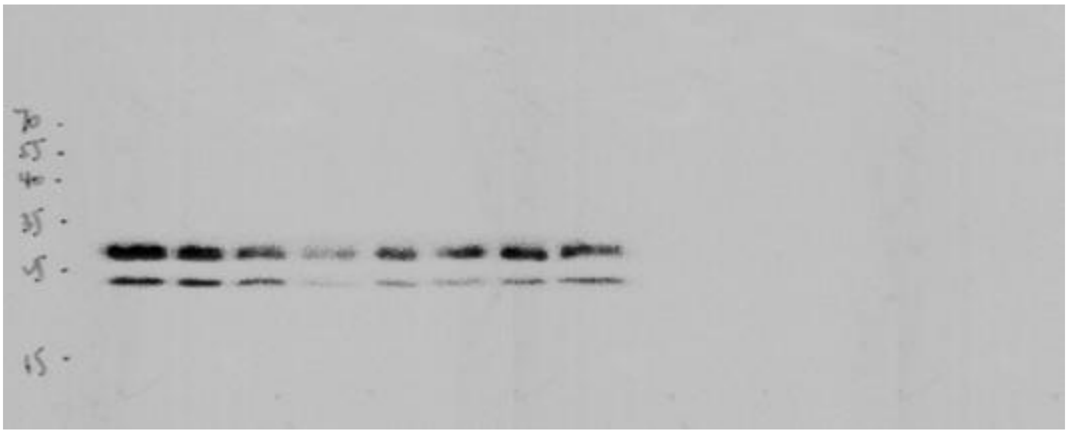

Fig5C. The expression of TMUB1 in the M phase and G1/S phase.

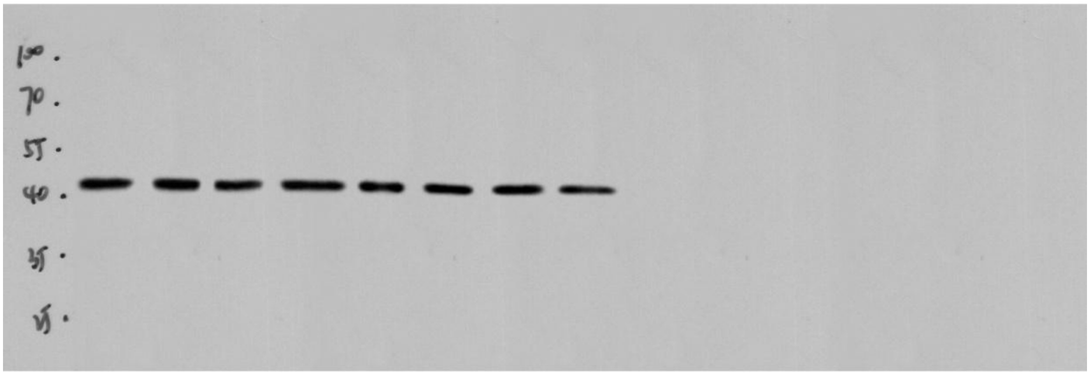

Fig5D. The expression of β -actin in the M phase and G1/S phase.

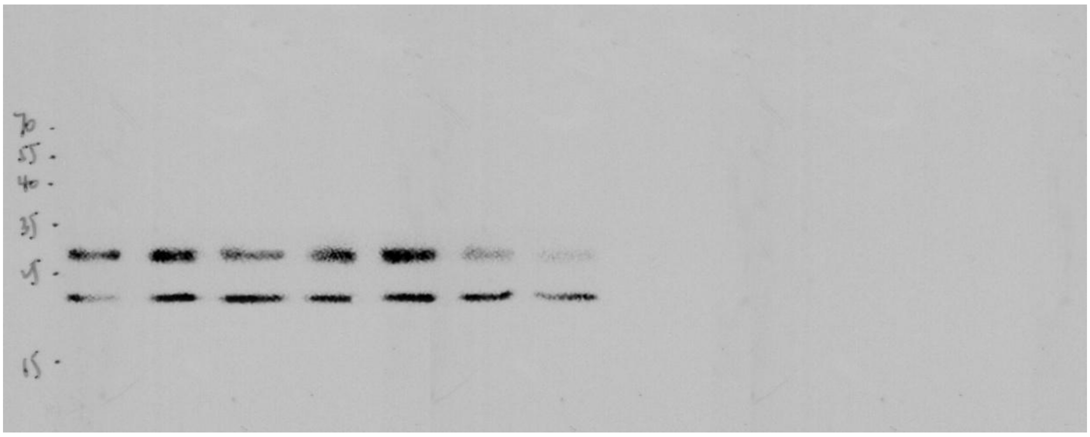

Fig5E. The expression of TMUB1 in the G0/G1 phase.

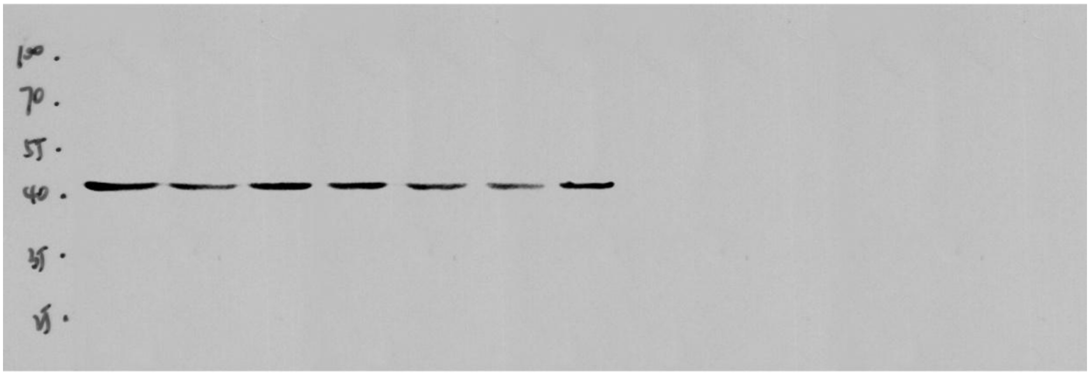

Fig5F. The expression of β -actin in the G0/G1 phase.

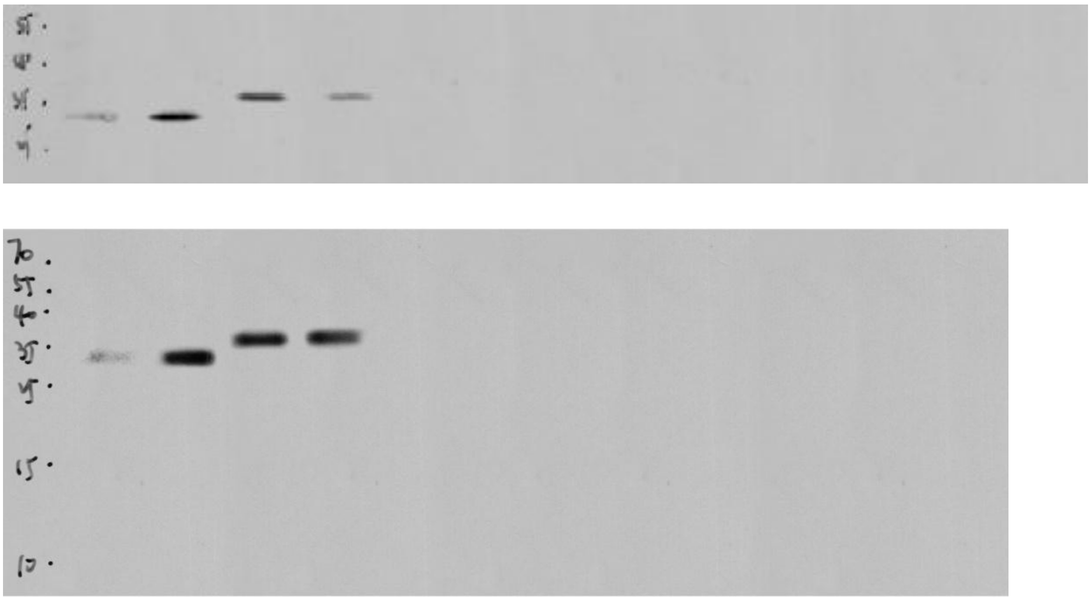

Fig5G. The effects of transfection of full-length TMUB1, its mutants and siRNA were tested by Westernblotting

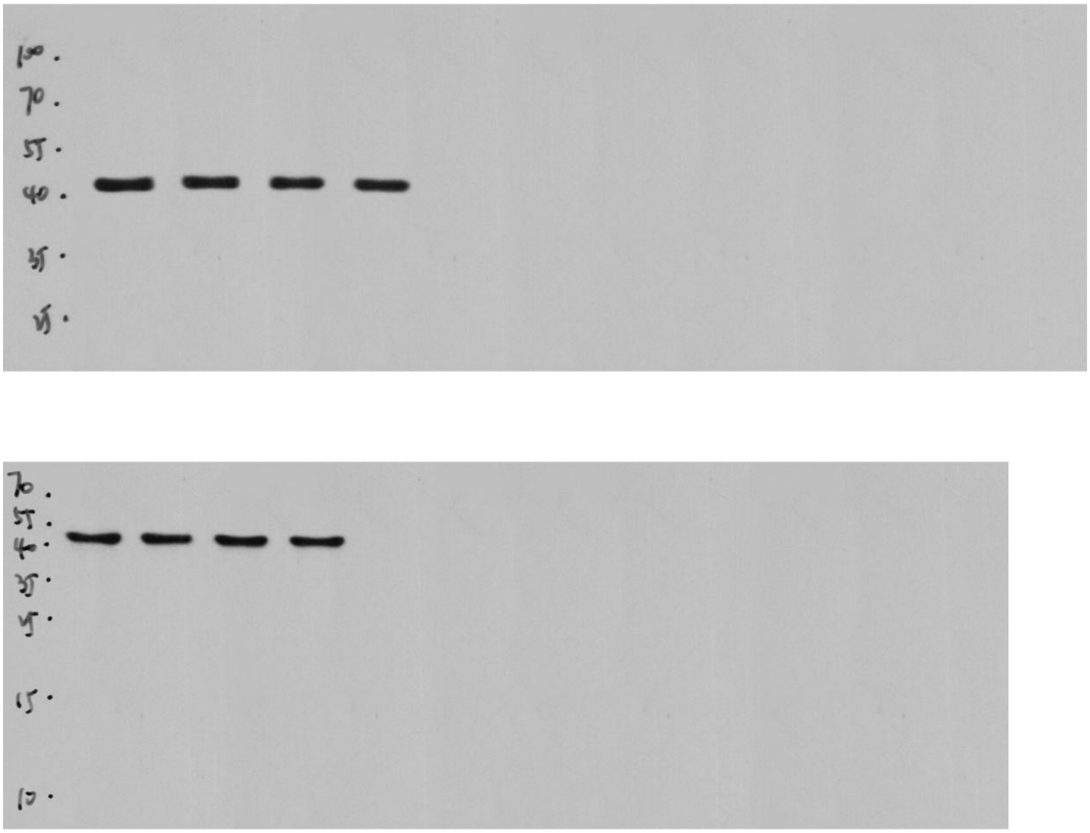

Fig5H. The expression of TMUB1 after transfection of full-length TMUB1, its mutants and siRNA were tested by Westernblotting

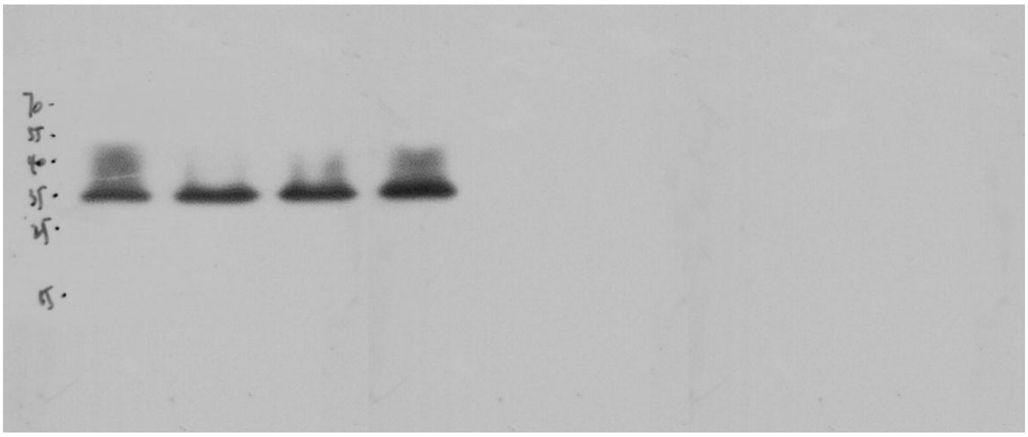

Fig5I. The expression of CAML after transfection of full-length TMUB1, its mutants and siRNA were tested by Westernblotting.

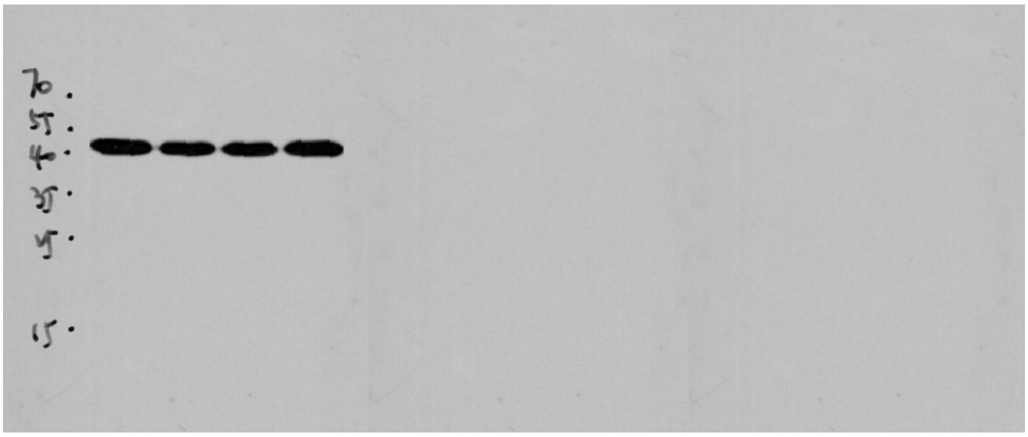

Fig5J. The expression of β -actin after transfection of full-length TMUB1, its mutants and siRNA were tested by Westernblotting.
